# Supplementary material for: Functional landscape of ubiquitin linkages couples K29-linked ubiquitylation to epigenome integrity
Source: EMBO J. 2025 Oct 22;44(23):6944–78. doi: 10.1038/s44318-025-00599-7 (PMC12669653; doi:10.1038/s44318-025-00599-7)
Supplement: Supplementary file 1 — Appendix [file 44318_2025_599_MOESM1_ESM.pdf]

Appendix for:

Functional landscape of ubiquitin linkages couples K29-linked ubiquitylation to epigenome integrity

Javier Arroyo-Gomez[1], Matthew J. Murray[1,2], Claire Guérillon[1,2], Juanjuan Wang[1], Ekaterina Isaakova[3], Nazaret Reverón-Gómez[1,2], Mikaela Koutrouli[1], Aldwin Suryo Rahmanto[3], Katrine Mitrofanov[1], Andreas Ingham[1], Sofie Schovsbo[1], Katrine Weischenfeldt[1,2], Fabian Coscia[1,4], Dimitris Typas[1], Moritz Völker-Albert[5], Victor Solis[5], Lars Juhl Jensen[1], Anja Groth[1,2], Andreas Mund[1], Petra Beli[3], Robert F Shearer[1], and Niels Mailand[1,2]

[1]Novo Nordisk Foundation Center for Protein Research, University of Copenhagen, 2200 Copenhagen, Denmark; [2]Danish Cancer Institute, Danish Cancer Society, Copenhagen, 2100 Copenhagen, Denmark; [3]Institute of Molecular Biology, 55218 Mainz, Germany; [4]Max Delbrück Center for Molecular Medicine, Berlin, Germany; [5]EpiQMAx GmbH, Planegg, Germany

Table of contents

Appendix Figure S1 Page 3

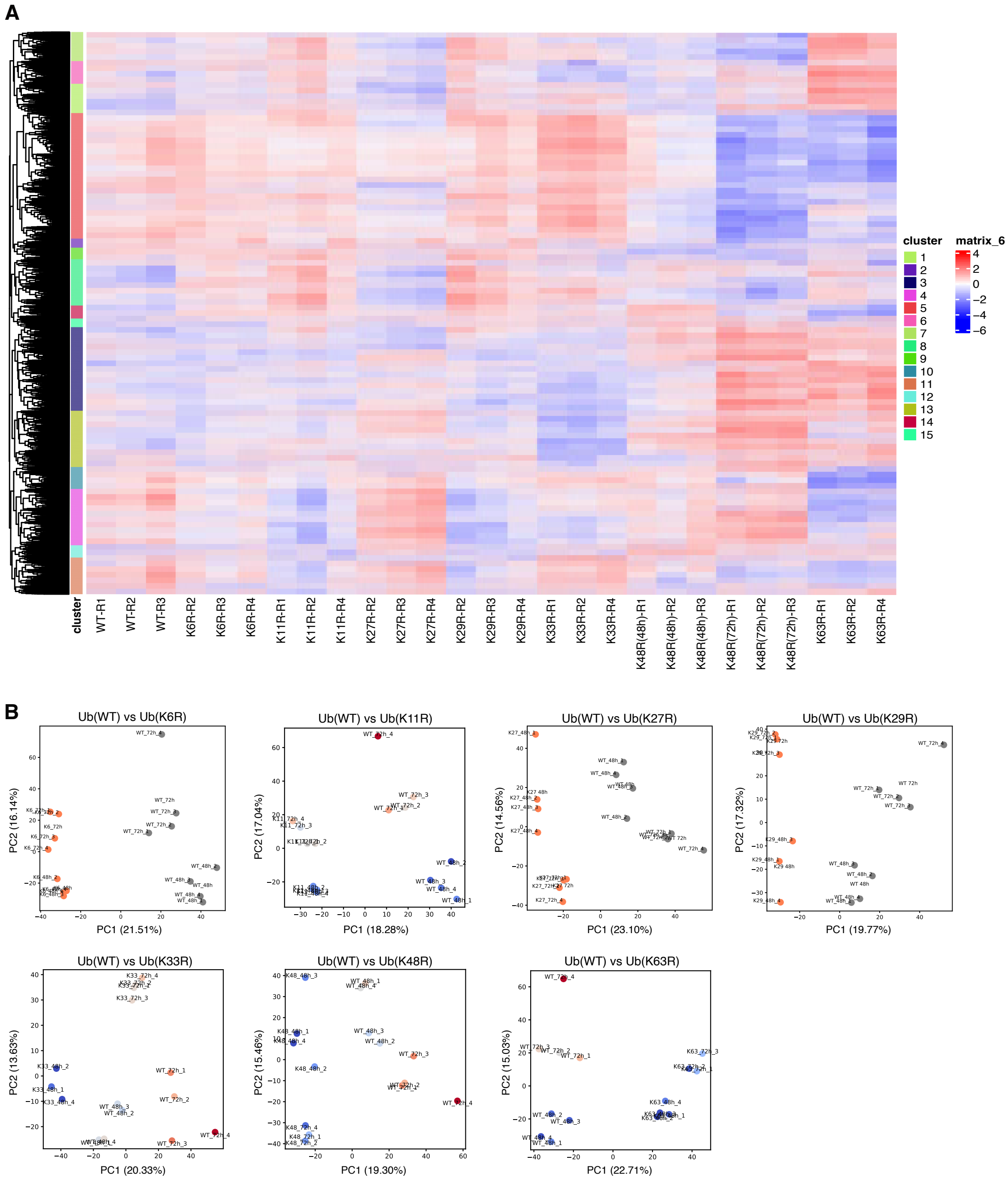

Appendix Figure S1.
Extended data related to Figure 2

A. Heatmap comparison of whole proteome analyses of individual replicates from indicated Ub replacement cell lines treated with DOX for 72 h (48 or 72 h in the case of Ub(K48R cells). See Table EV2 for full results.
B. Principal component analysis clustering of samples analyzed by MS after enrichment of HA-Ub conjugates from Ub replacement cell lines under denaturing conditions (Figure 2A-H).
