# Supplementary figures and images for: Functional landscape of ubiquitin linkages couples K29-linked ubiquitylation to epigenome integrity
Source: EMBO J. 2025 Oct 22;44(23):6944–78. doi: 10.1038/s44318-025-00599-7 (PMC12669653; doi:10.1038/s44318-025-00599-7)

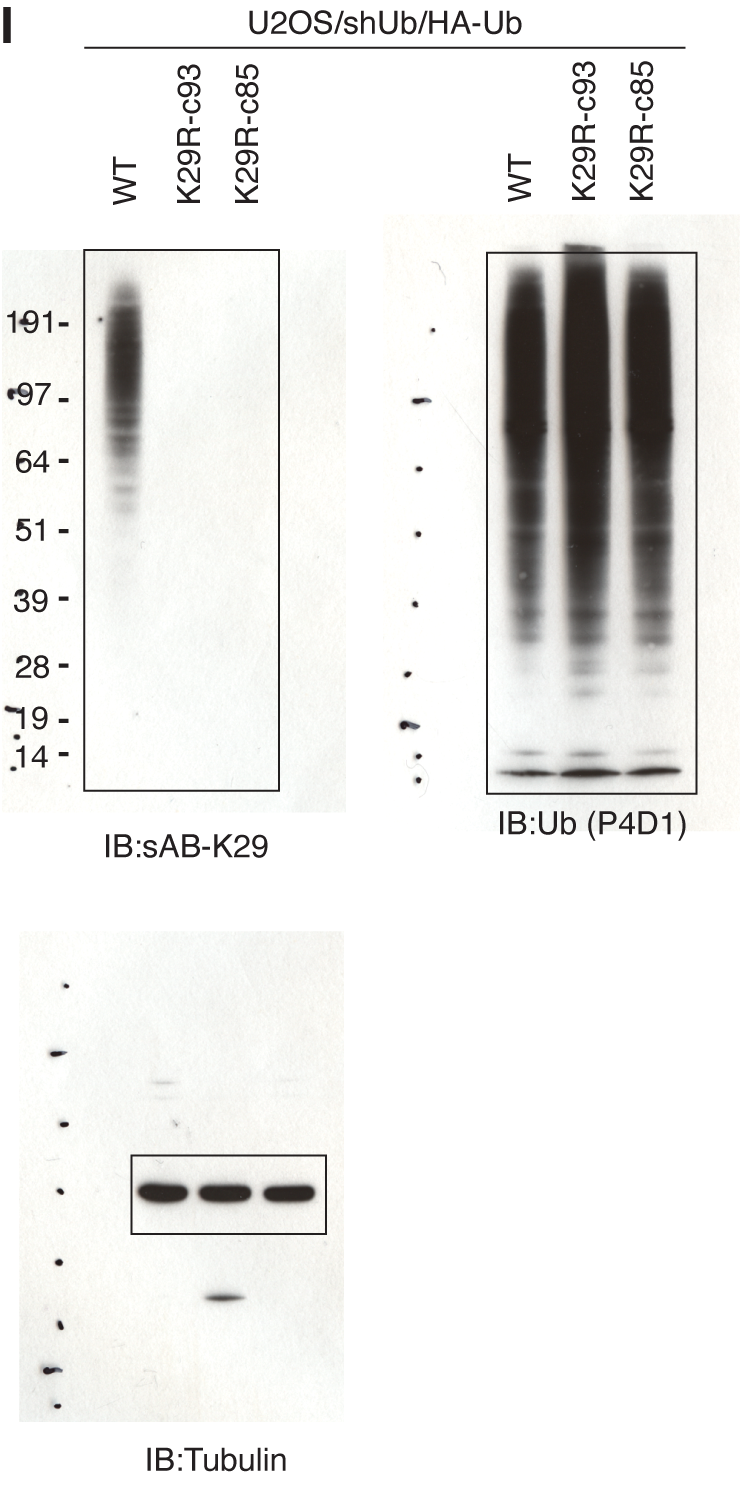

Supplement: Supplementary file 8 — Source data Fig. 1 [file 44318_2025_599_MOESM8_ESM.zip › Figure 1/1I/blot images.tif]

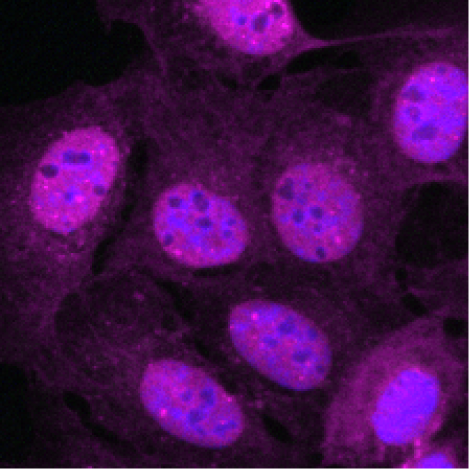

Supplement: Supplementary file 8 — Source data Fig. 1 [file 44318_2025_599_MOESM8_ESM.zip › Figure 1/1D/shUb HAUb no Dox merge.tif]

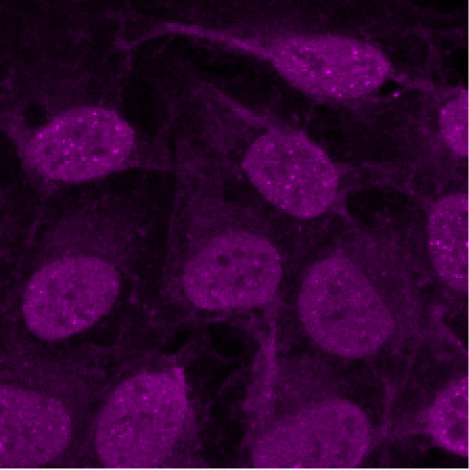

Supplement: Supplementary file 8 — Source data Fig. 1 [file 44318_2025_599_MOESM8_ESM.zip › Figure 1/1D/shUb no Dox FK2.tif]

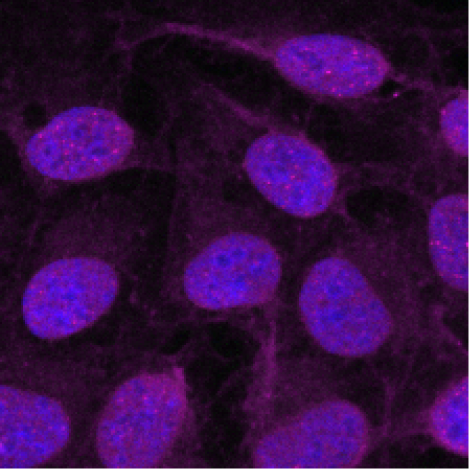

Supplement: Supplementary file 8 — Source data Fig. 1 [file 44318_2025_599_MOESM8_ESM.zip › Figure 1/1D/shUb no Dox merge.tif]

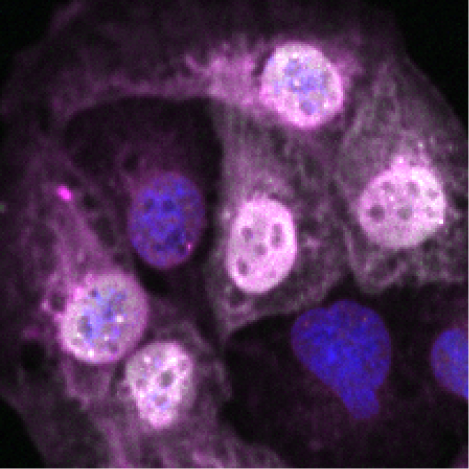

Supplement: Supplementary file 8 — Source data Fig. 1 [file 44318_2025_599_MOESM8_ESM.zip › Figure 1/1D/shUb HAUb Dox merge.tif]

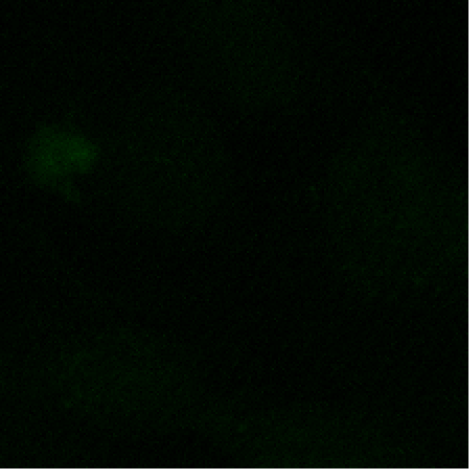

Supplement: Supplementary file 8 — Source data Fig. 1 [file 44318_2025_599_MOESM8_ESM.zip › Figure 1/1D/shUb Dox HA.tif]

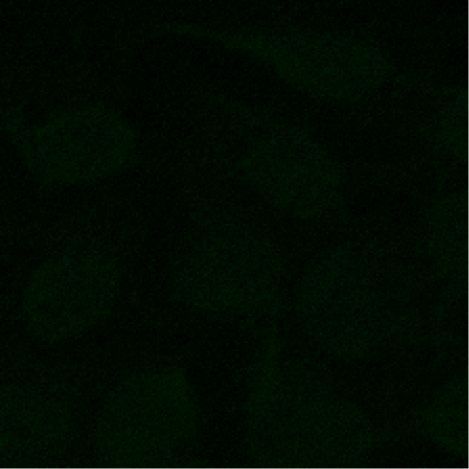

Supplement: Supplementary file 8 — Source data Fig. 1 [file 44318_2025_599_MOESM8_ESM.zip › Figure 1/1D/shUb no Dox HA.tif]

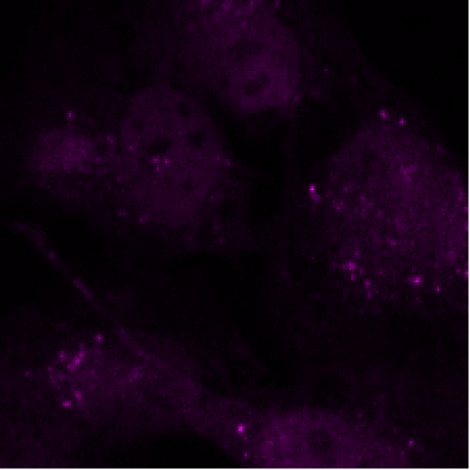

Supplement: Supplementary file 8 — Source data Fig. 1 [file 44318_2025_599_MOESM8_ESM.zip › Figure 1/1D/shUb Dox FK2.tif]

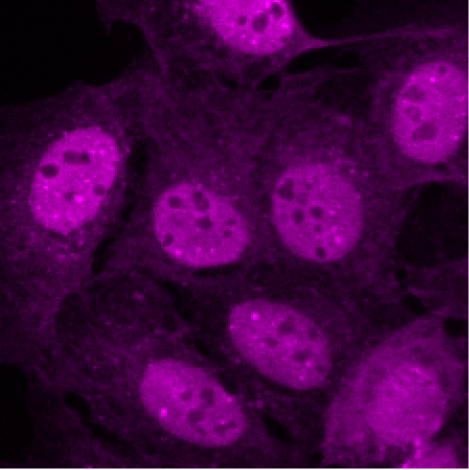

Supplement: Supplementary file 8 — Source data Fig. 1 [file 44318_2025_599_MOESM8_ESM.zip › Figure 1/1D/shUb HAUb no Dox FK2.tif]

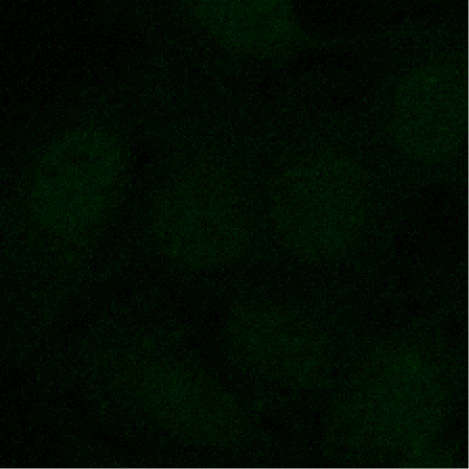

Supplement: Supplementary file 8 — Source data Fig. 1 [file 44318_2025_599_MOESM8_ESM.zip › Figure 1/1D/shUb HAUb no Dox HA.tif]

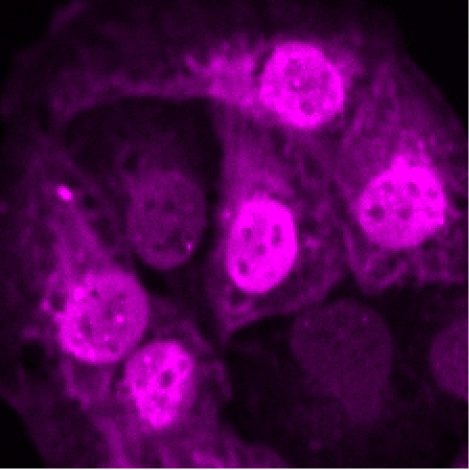

Supplement: Supplementary file 8 — Source data Fig. 1 [file 44318_2025_599_MOESM8_ESM.zip › Figure 1/1D/shUb HAUb Dox FK2.tif]

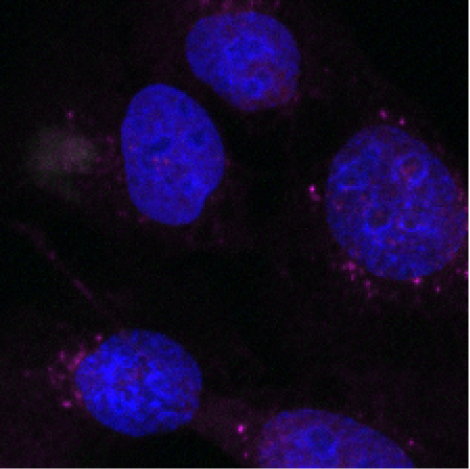

Supplement: Supplementary file 8 — Source data Fig. 1 [file 44318_2025_599_MOESM8_ESM.zip › Figure 1/1D/shUb Dox merge.tif]

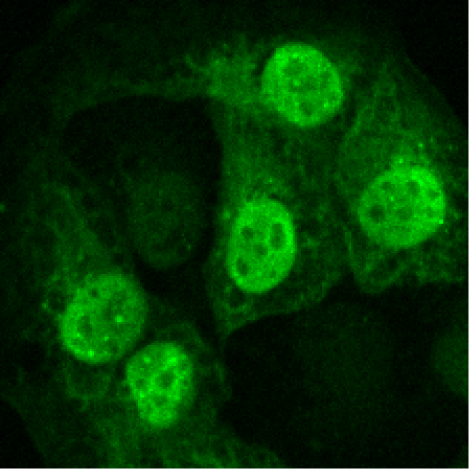

Supplement: Supplementary file 8 — Source data Fig. 1 [file 44318_2025_599_MOESM8_ESM.zip › Figure 1/1D/shUb HAUb Dox HA.tif]

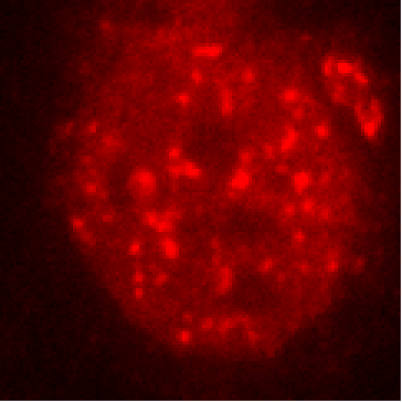

Supplement: Supplementary file 8 — Source data Fig. 1 [file 44318_2025_599_MOESM8_ESM.zip › Figure 1/1J/WT FK2.tif]

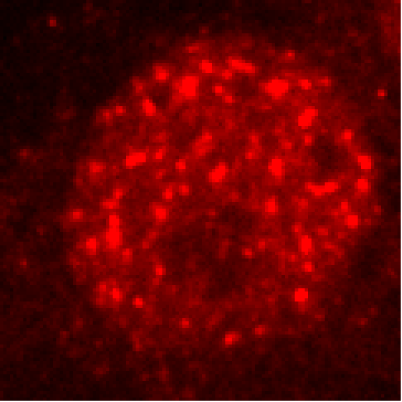

Supplement: Supplementary file 8 — Source data Fig. 1 [file 44318_2025_599_MOESM8_ESM.zip › Figure 1/1J/K6R FK2.tif]

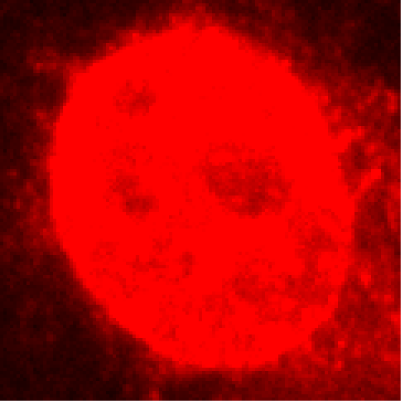

Supplement: Supplementary file 8 — Source data Fig. 1 [file 44318_2025_599_MOESM8_ESM.zip › Figure 1/1J/K48R FK2.tif]

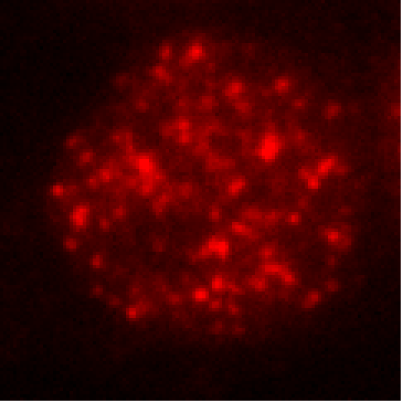

Supplement: Supplementary file 8 — Source data Fig. 1 [file 44318_2025_599_MOESM8_ESM.zip › Figure 1/1J/K11R H2AX.tif]

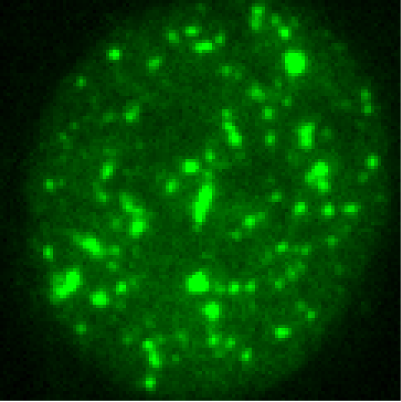

Supplement: Supplementary file 8 — Source data Fig. 1 [file 44318_2025_599_MOESM8_ESM.zip › Figure 1/1J/K29R 53BP1.tif]

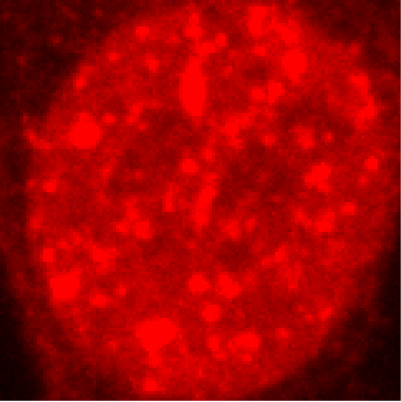

Supplement: Supplementary file 8 — Source data Fig. 1 [file 44318_2025_599_MOESM8_ESM.zip › Figure 1/1J/K29R FK2.tif]

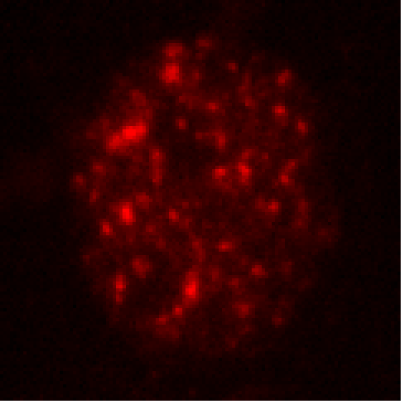

Supplement: Supplementary file 8 — Source data Fig. 1 [file 44318_2025_599_MOESM8_ESM.zip › Figure 1/1J/K29R H2AX.tif]

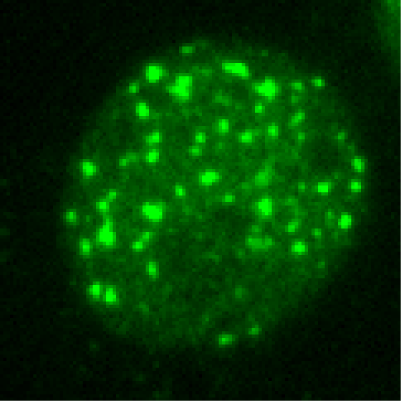

Supplement: Supplementary file 8 — Source data Fig. 1 [file 44318_2025_599_MOESM8_ESM.zip › Figure 1/1J/K6R 53BP1.tif]

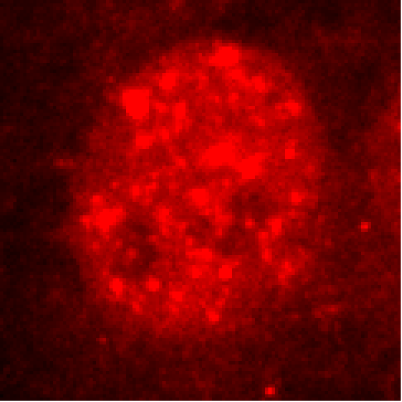

Supplement: Supplementary file 8 — Source data Fig. 1 [file 44318_2025_599_MOESM8_ESM.zip › Figure 1/1J/K11R FK2.tif]

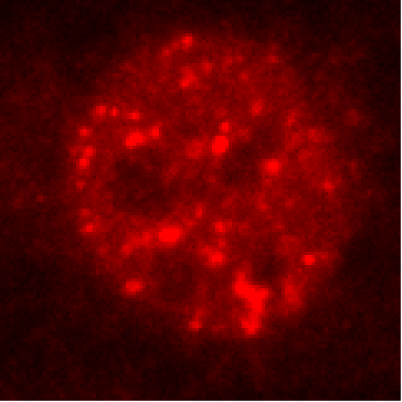

Supplement: Supplementary file 8 — Source data Fig. 1 [file 44318_2025_599_MOESM8_ESM.zip › Figure 1/1J/K33R FK2.tif]

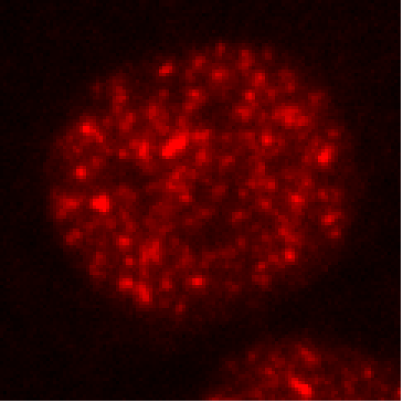

Supplement: Supplementary file 8 — Source data Fig. 1 [file 44318_2025_599_MOESM8_ESM.zip › Figure 1/1J/WT H2AX.tif]

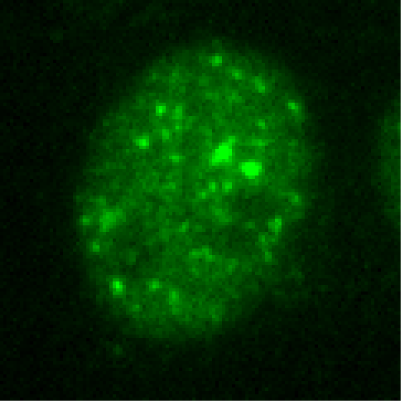

Supplement: Supplementary file 8 — Source data Fig. 1 [file 44318_2025_599_MOESM8_ESM.zip › Figure 1/1J/K11R 53BP1.tif]

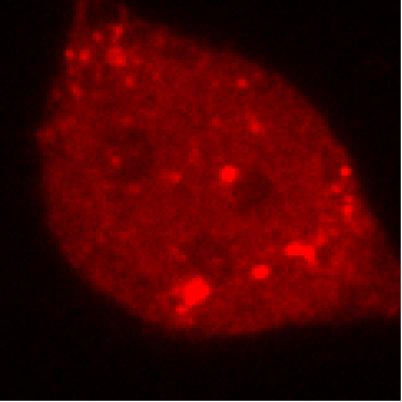

Supplement: Supplementary file 8 — Source data Fig. 1 [file 44318_2025_599_MOESM8_ESM.zip › Figure 1/1J/K63R FK2.tif]

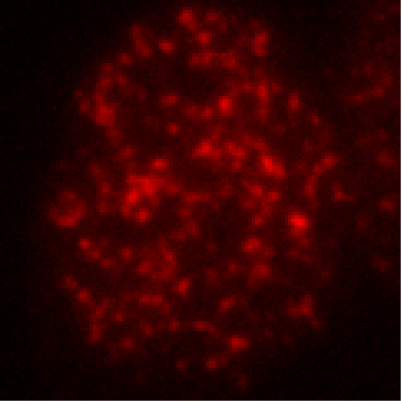

Supplement: Supplementary file 8 — Source data Fig. 1 [file 44318_2025_599_MOESM8_ESM.zip › Figure 1/1J/K48R H2AX.tif]

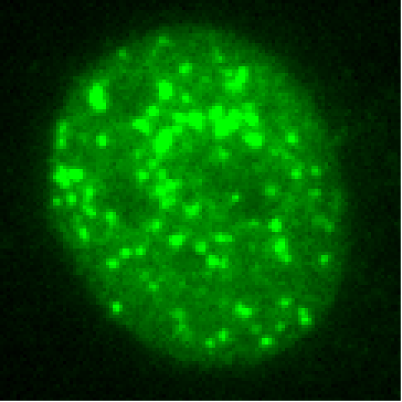

Supplement: Supplementary file 8 — Source data Fig. 1 [file 44318_2025_599_MOESM8_ESM.zip › Figure 1/1J/K48R 53BP1.tif]

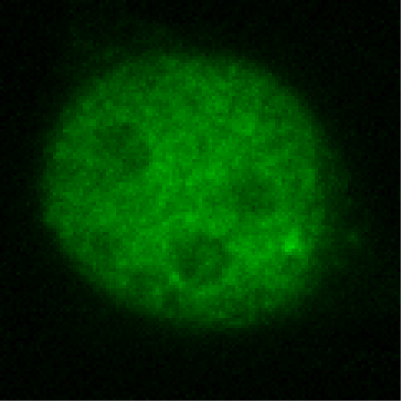

Supplement: Supplementary file 8 — Source data Fig. 1 [file 44318_2025_599_MOESM8_ESM.zip › Figure 1/1J/K63R 53BP1.tif]

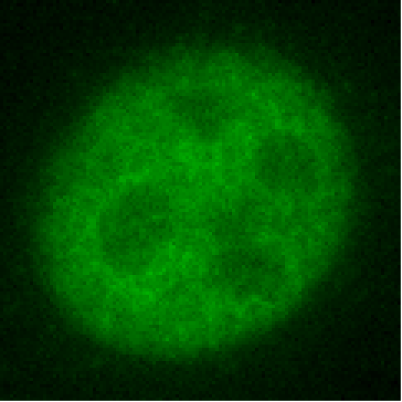

Supplement: Supplementary file 8 — Source data Fig. 1 [file 44318_2025_599_MOESM8_ESM.zip › Figure 1/1J/WTE1i 53BP1.tif]

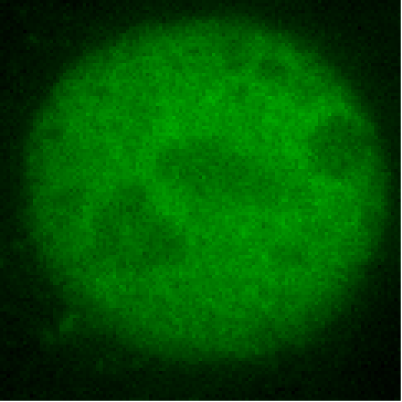

Supplement: Supplementary file 8 — Source data Fig. 1 [file 44318_2025_599_MOESM8_ESM.zip › Figure 1/1J/K27R 53BP1.tif]

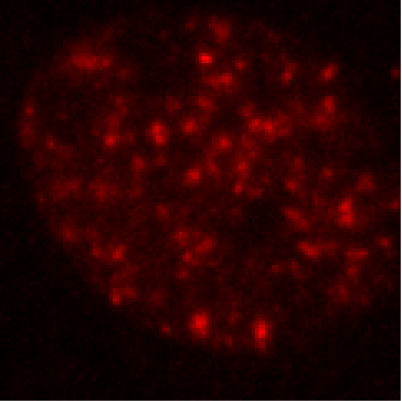

Supplement: Supplementary file 8 — Source data Fig. 1 [file 44318_2025_599_MOESM8_ESM.zip › Figure 1/1J/K63R H2AX.tif]

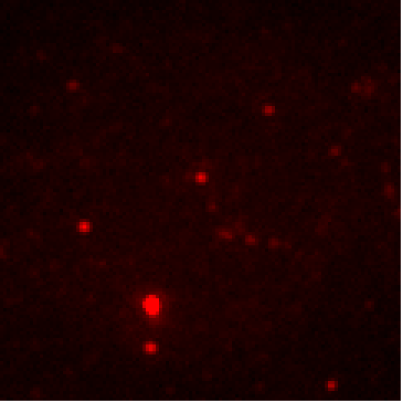

Supplement: Supplementary file 8 — Source data Fig. 1 [file 44318_2025_599_MOESM8_ESM.zip › Figure 1/1J/WTE1i FK2.tif]

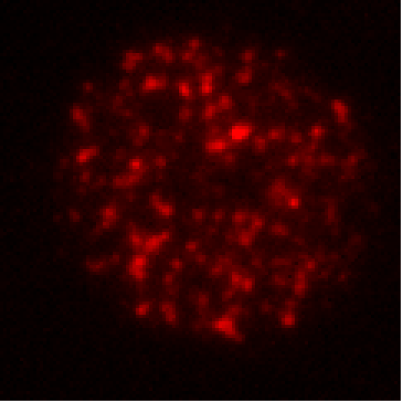

Supplement: Supplementary file 8 — Source data Fig. 1 [file 44318_2025_599_MOESM8_ESM.zip › Figure 1/1J/K33R H2AX.tif]

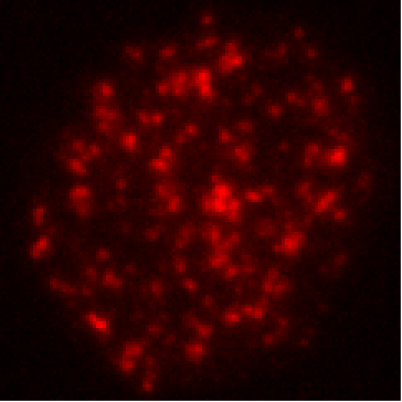

Supplement: Supplementary file 8 — Source data Fig. 1 [file 44318_2025_599_MOESM8_ESM.zip › Figure 1/1J/K27R H2AX.tif]

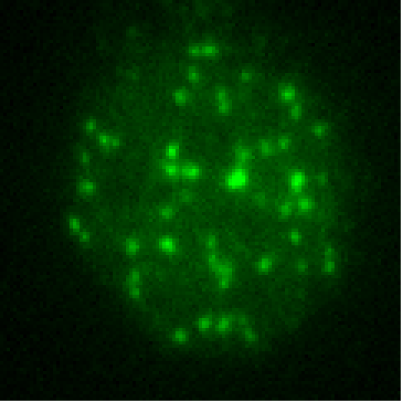

Supplement: Supplementary file 8 — Source data Fig. 1 [file 44318_2025_599_MOESM8_ESM.zip › Figure 1/1J/WT 53BP1.tif]

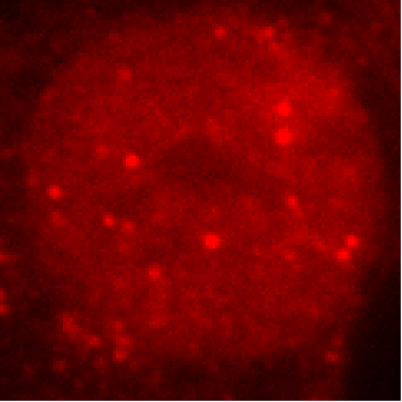

Supplement: Supplementary file 8 — Source data Fig. 1 [file 44318_2025_599_MOESM8_ESM.zip › Figure 1/1J/K27R FK2.tif]

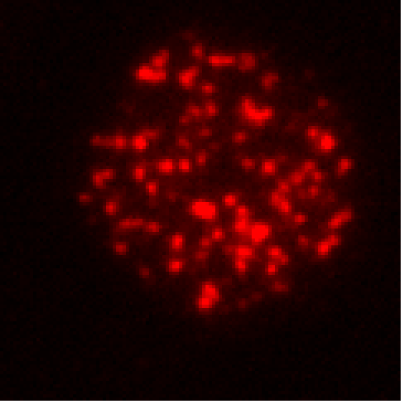

Supplement: Supplementary file 8 — Source data Fig. 1 [file 44318_2025_599_MOESM8_ESM.zip › Figure 1/1J/WTE1i H2AX.tif]

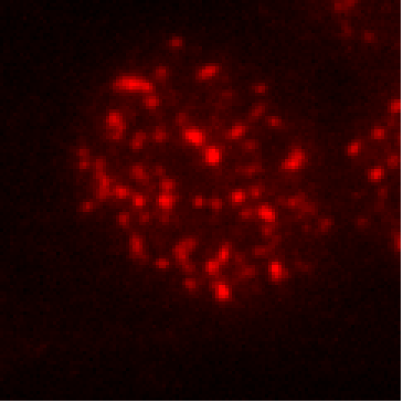

Supplement: Supplementary file 8 — Source data Fig. 1 [file 44318_2025_599_MOESM8_ESM.zip › Figure 1/1J/K6R H2AX.tif]

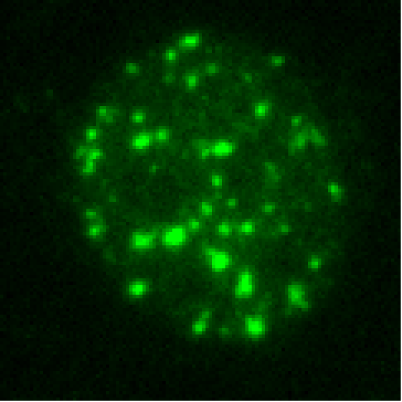

Supplement: Supplementary file 8 — Source data Fig. 1 [file 44318_2025_599_MOESM8_ESM.zip › Figure 1/1J/K33R 53BP1.tif]

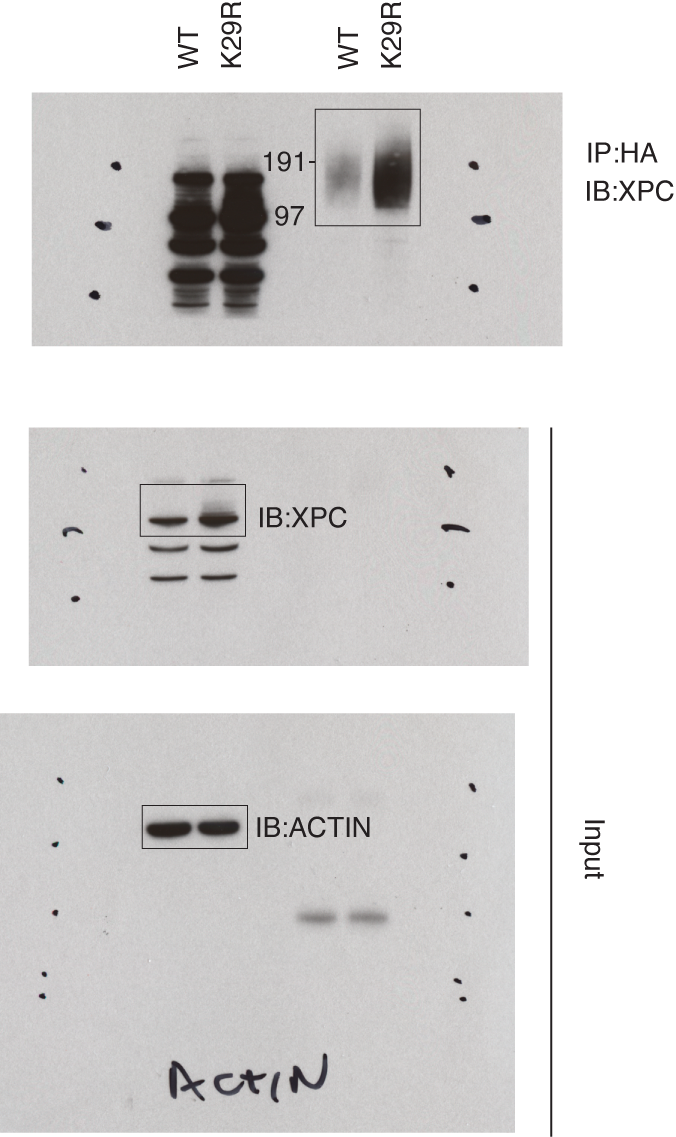

Supplement: Supplementary file 9 — Source data Fig. 2 [file 44318_2025_599_MOESM9_ESM.zip › Figure 2/2I/XPC blots.tif]

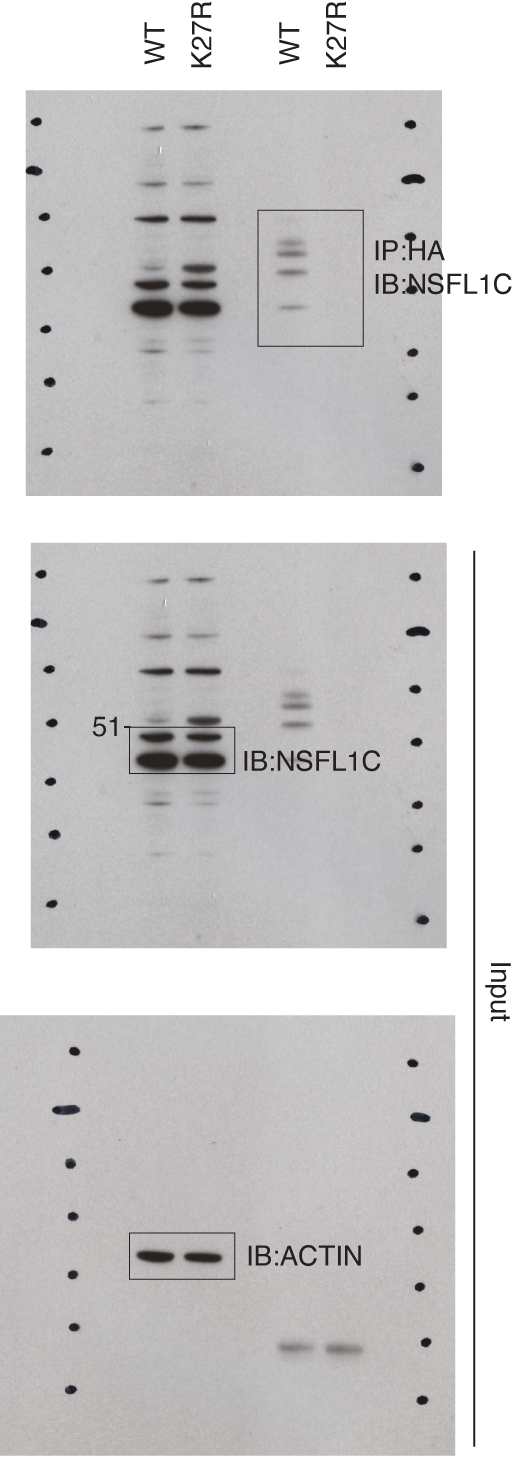

Supplement: Supplementary file 9 — Source data Fig. 2 [file 44318_2025_599_MOESM9_ESM.zip › Figure 2/2I/NSFL1C blots.tif]

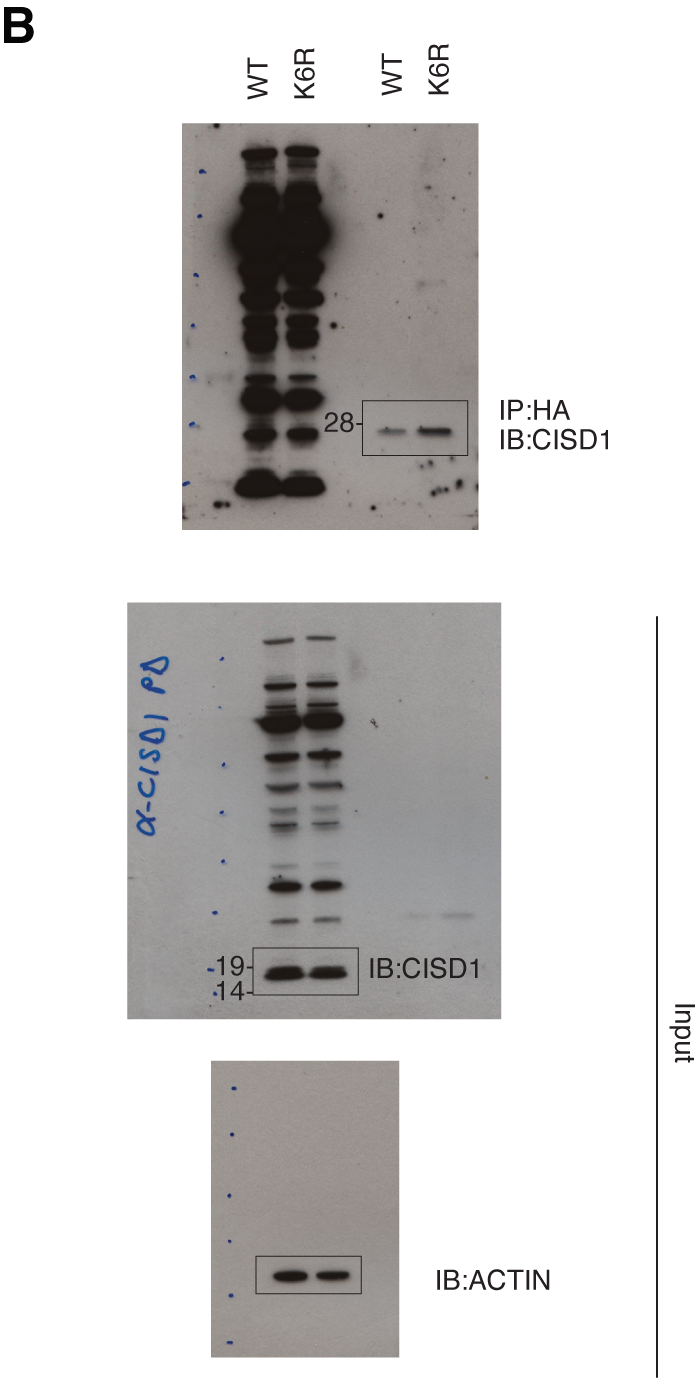

Supplement: Supplementary file 9 — Source data Fig. 2 [file 44318_2025_599_MOESM9_ESM.zip › Figure 2/2I/CISD1 blots.tif]

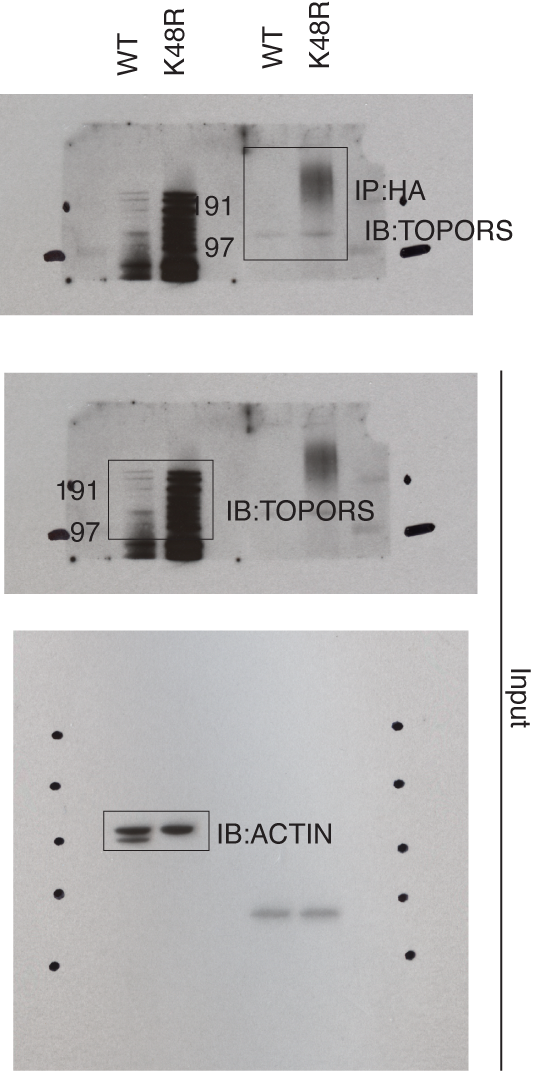

Supplement: Supplementary file 9 — Source data Fig. 2 [file 44318_2025_599_MOESM9_ESM.zip › Figure 2/2I/TOPORS blots.tif]

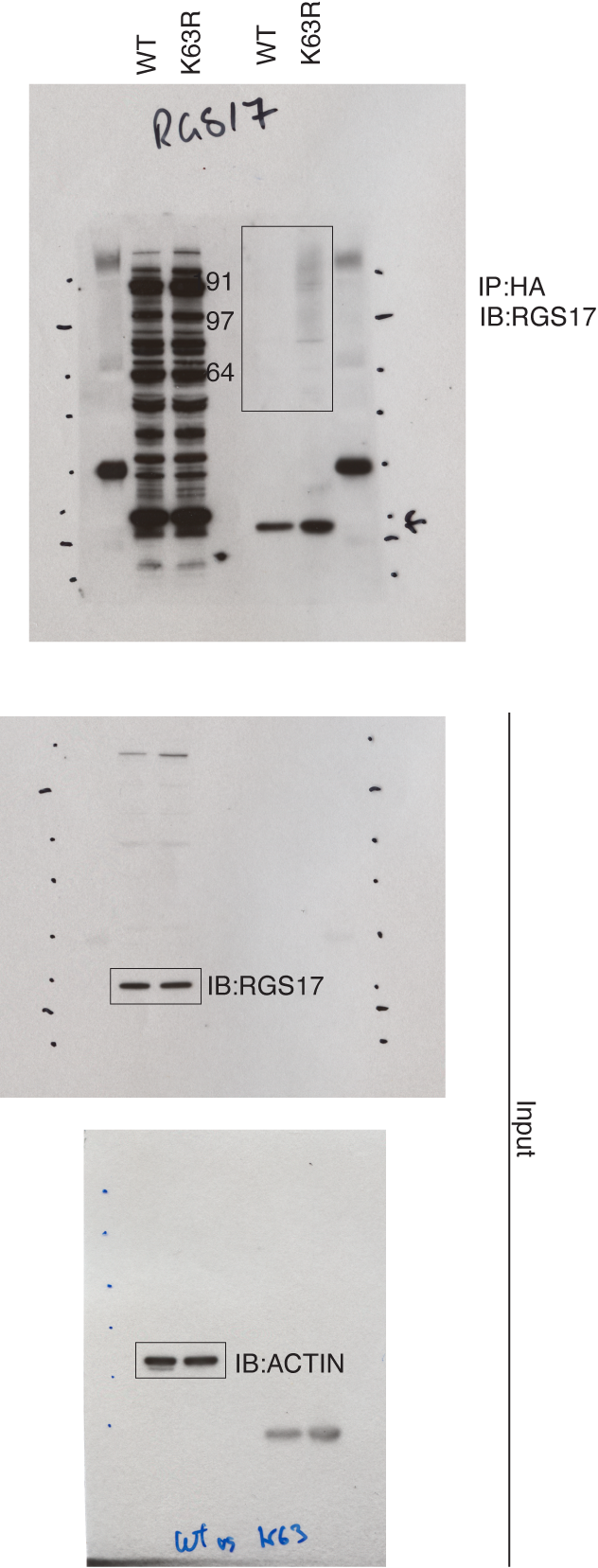

Supplement: Supplementary file 9 — Source data Fig. 2 [file 44318_2025_599_MOESM9_ESM.zip › Figure 2/2I/RGS17 blots.tif]

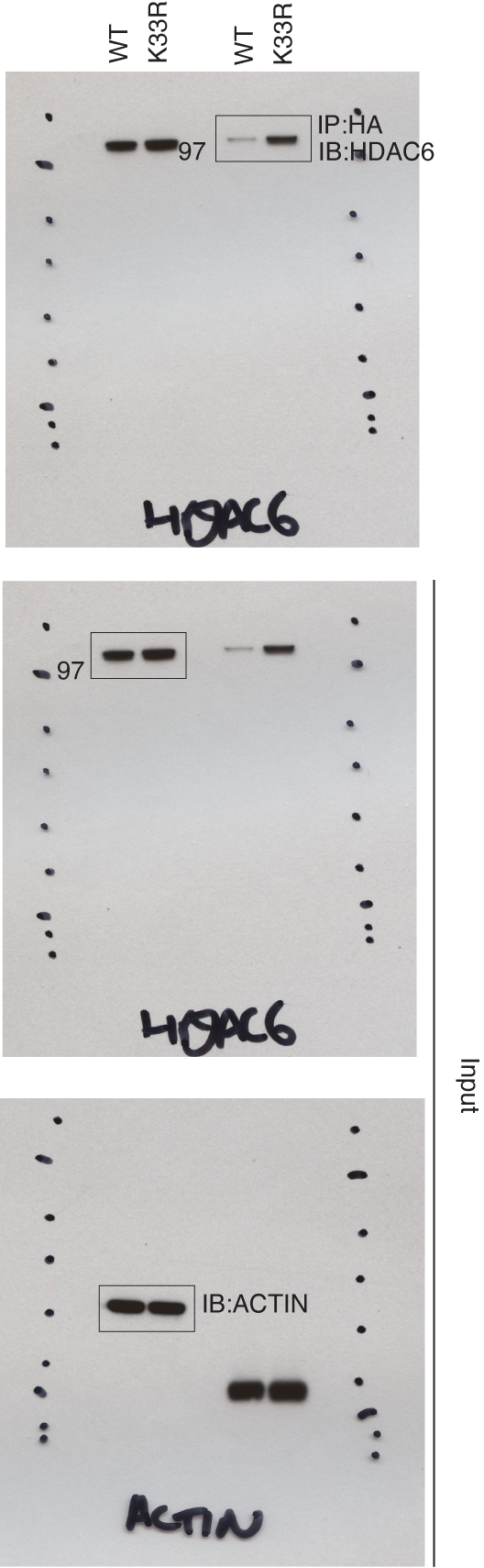

Supplement: Supplementary file 9 — Source data Fig. 2 [file 44318_2025_599_MOESM9_ESM.zip › Figure 2/2I/HDAC6 blots.tif]

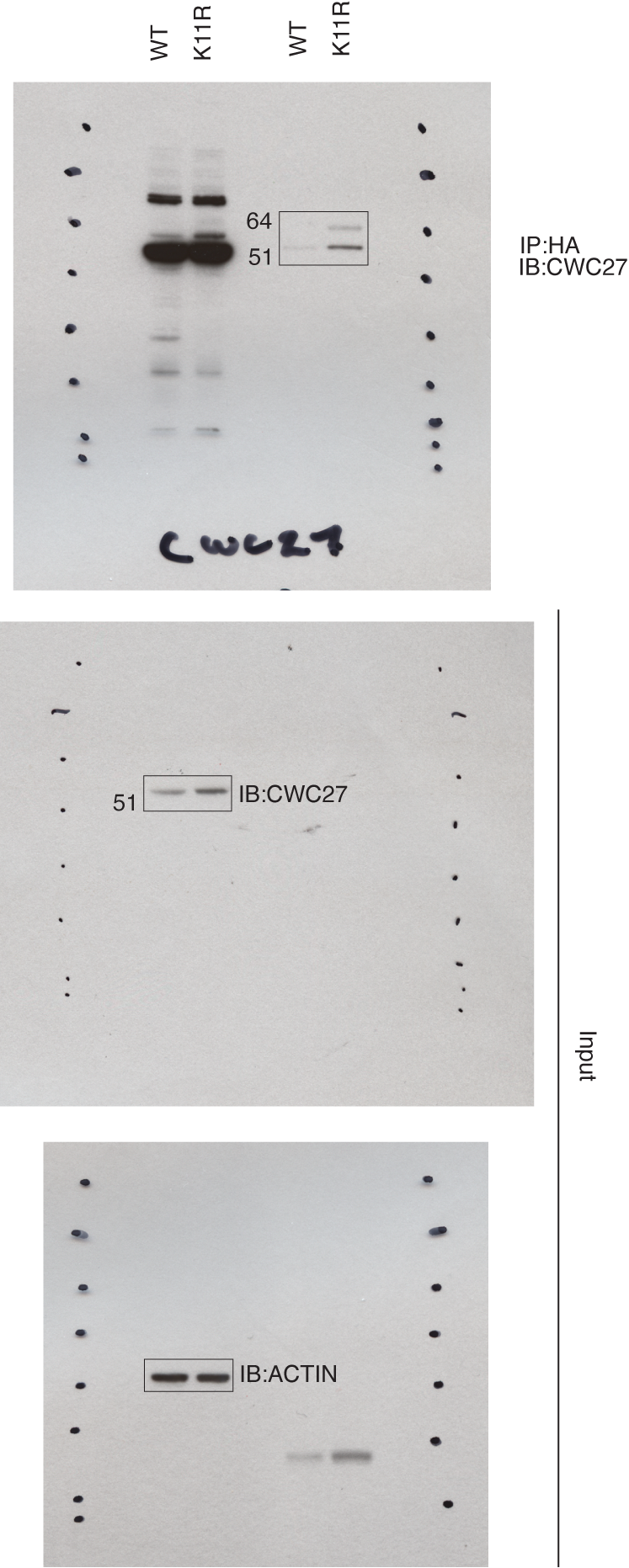

Supplement: Supplementary file 9 — Source data Fig. 2 [file 44318_2025_599_MOESM9_ESM.zip › Figure 2/2I/CWC27 blots.tif]

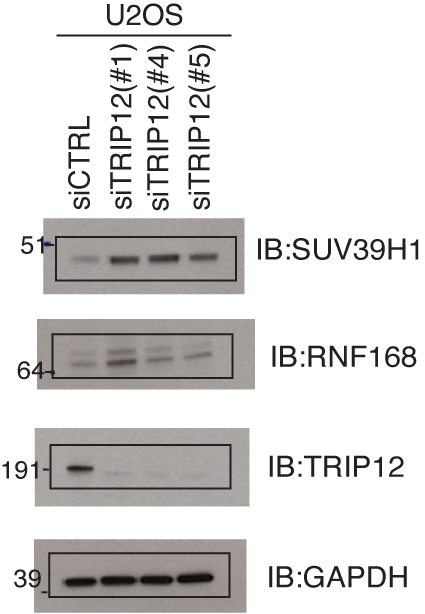

Supplement: Supplementary file 10 — Source data Fig. 3 [file 44318_2025_599_MOESM10_ESM.zip › Figure 3/3K/blots.tif]

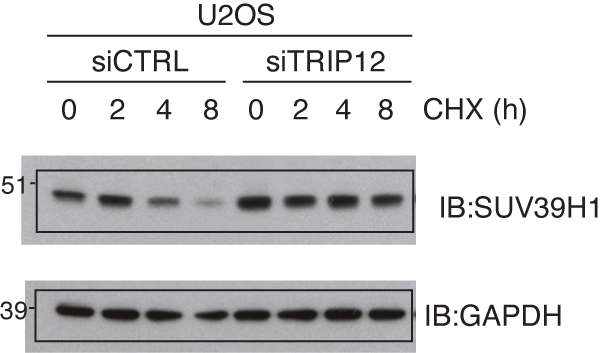

Supplement: Supplementary file 10 — Source data Fig. 3 [file 44318_2025_599_MOESM10_ESM.zip › Figure 3/3L/blots.tif]

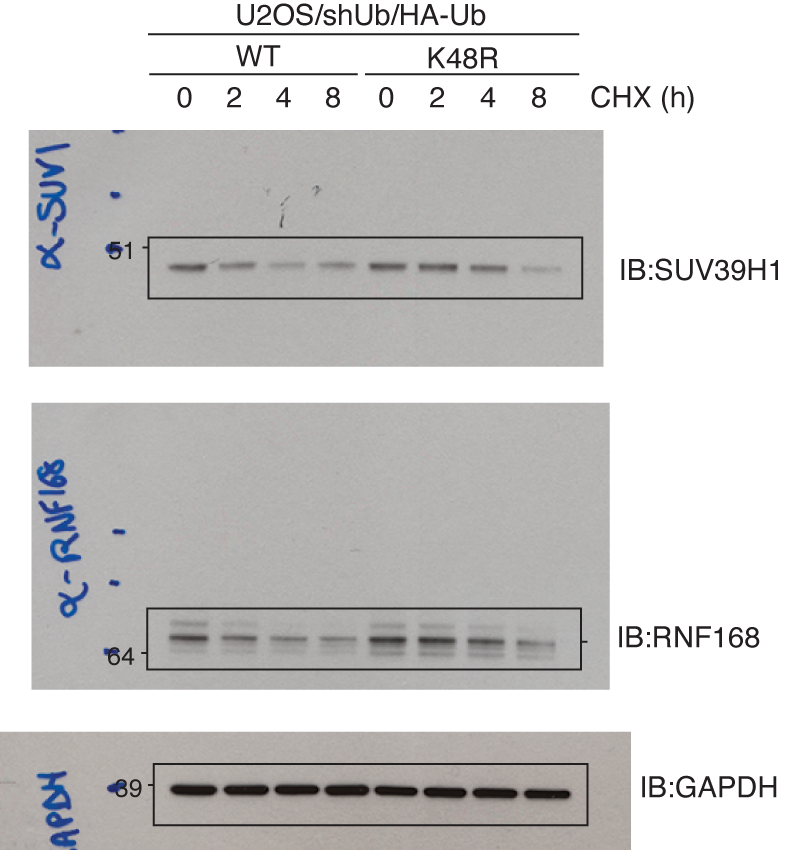

Supplement: Supplementary file 10 — Source data Fig. 3 [file 44318_2025_599_MOESM10_ESM.zip › Figure 3/3E/blots.tif]

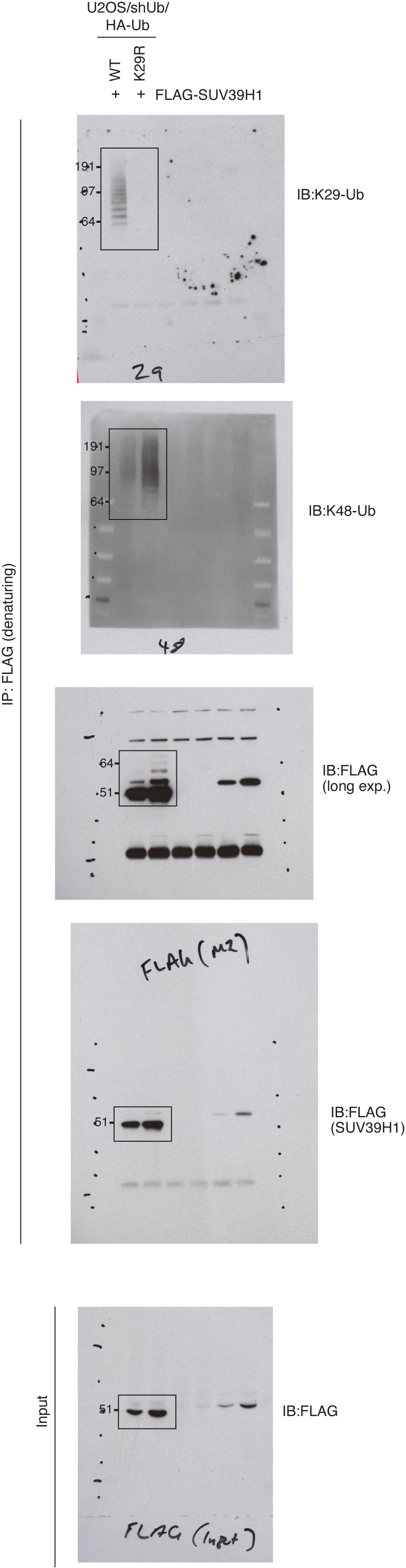

Supplement: Supplementary file 10 — Source data Fig. 3 [file 44318_2025_599_MOESM10_ESM.zip › Figure 3/3B/blots.tif]

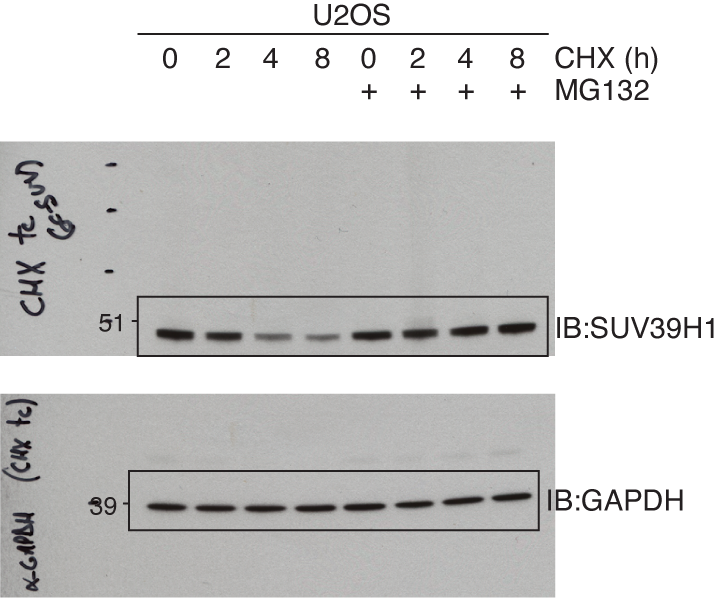

Supplement: Supplementary file 10 — Source data Fig. 3 [file 44318_2025_599_MOESM10_ESM.zip › Figure 3/3C/blots.tif]

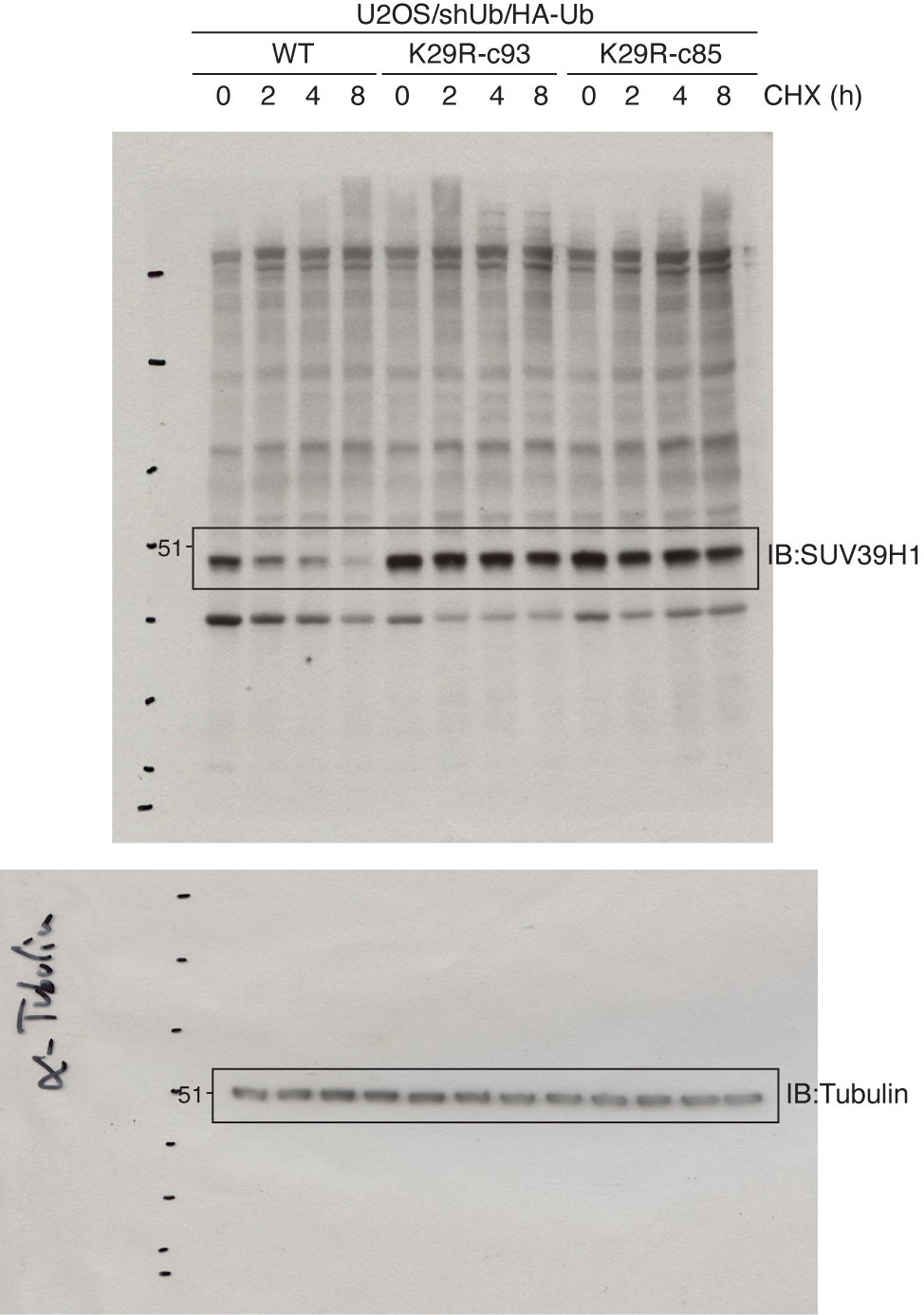

Supplement: Supplementary file 10 — Source data Fig. 3 [file 44318_2025_599_MOESM10_ESM.zip › Figure 3/3D/blots.tif]

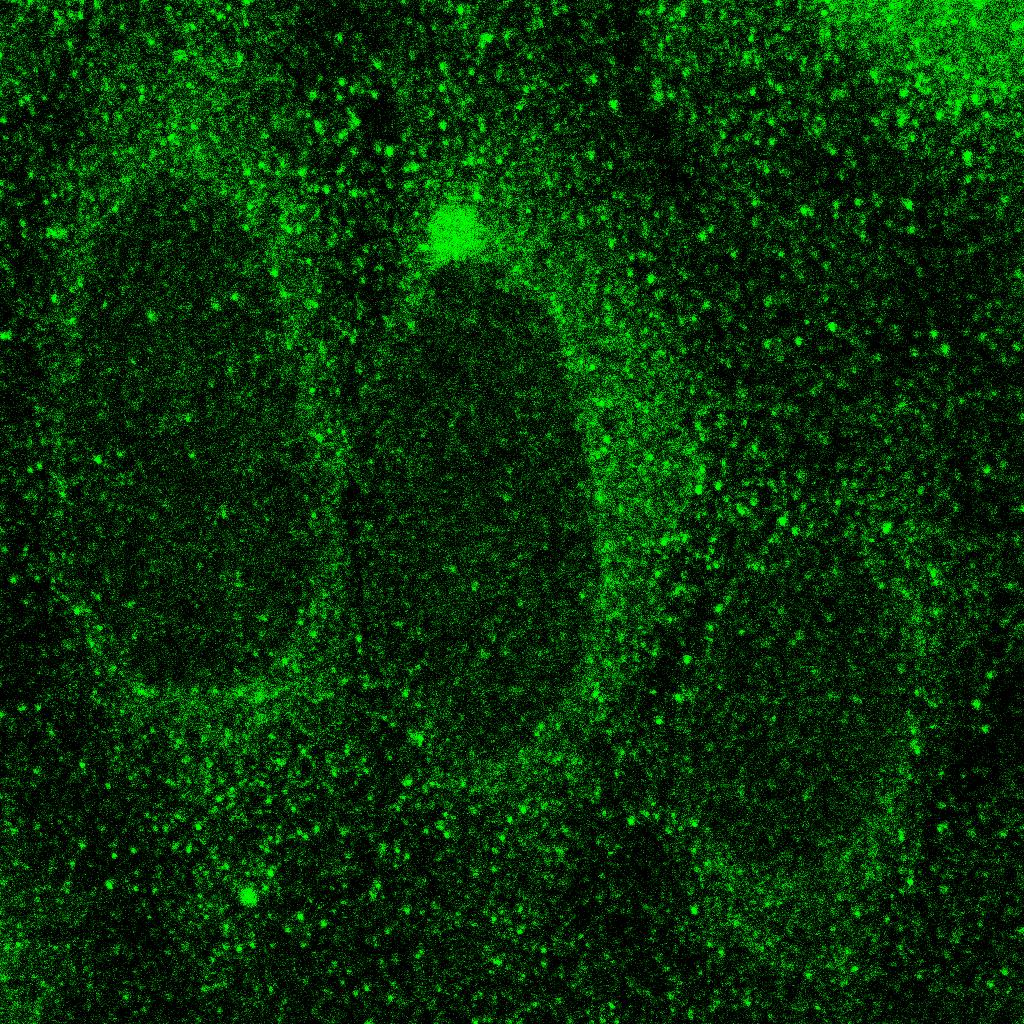

Supplement: Supplementary file 10 — Source data Fig. 3 [file 44318_2025_599_MOESM10_ESM.zip › Figure 3/3H/Fig 3H siTRIP12 sAB-K29.tif]

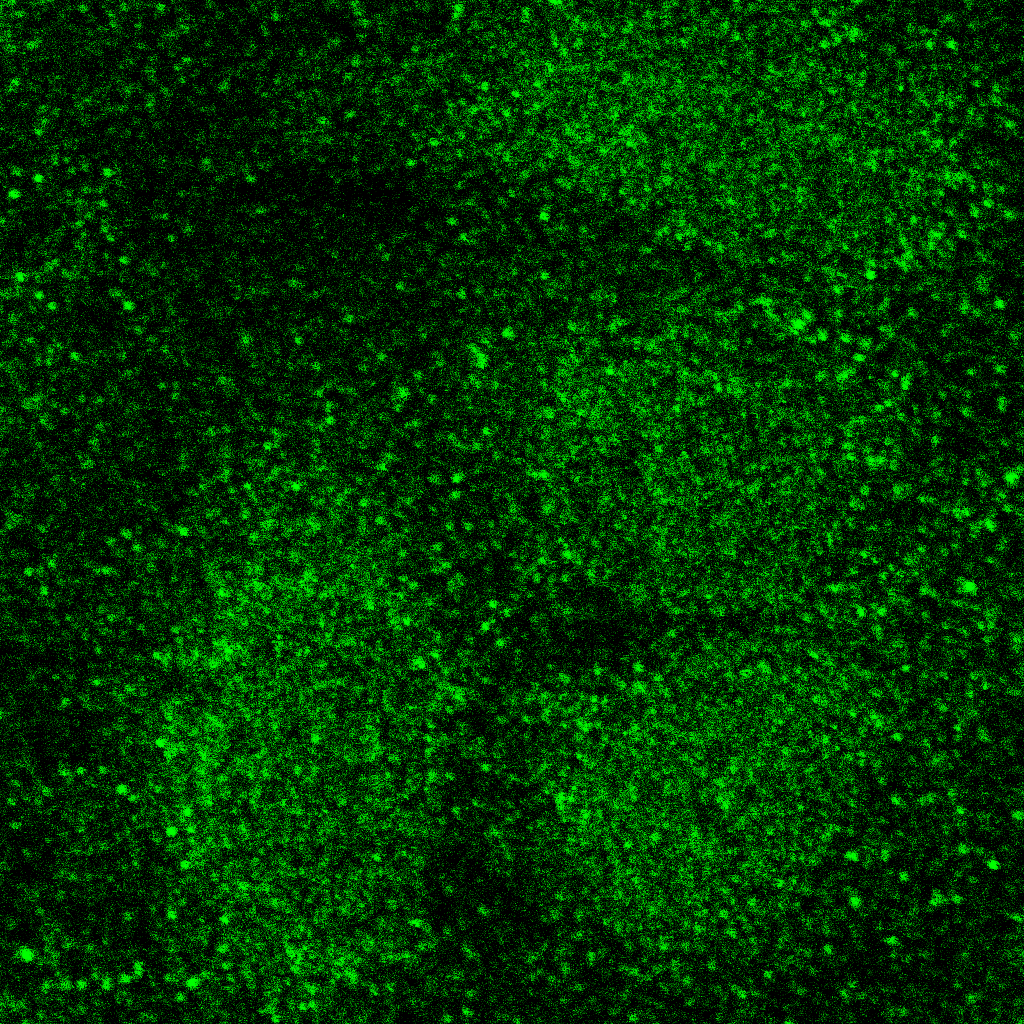

Supplement: Supplementary file 10 — Source data Fig. 3 [file 44318_2025_599_MOESM10_ESM.zip › Figure 3/3H/Fig 3H siCTRL sAB-K29.tif]

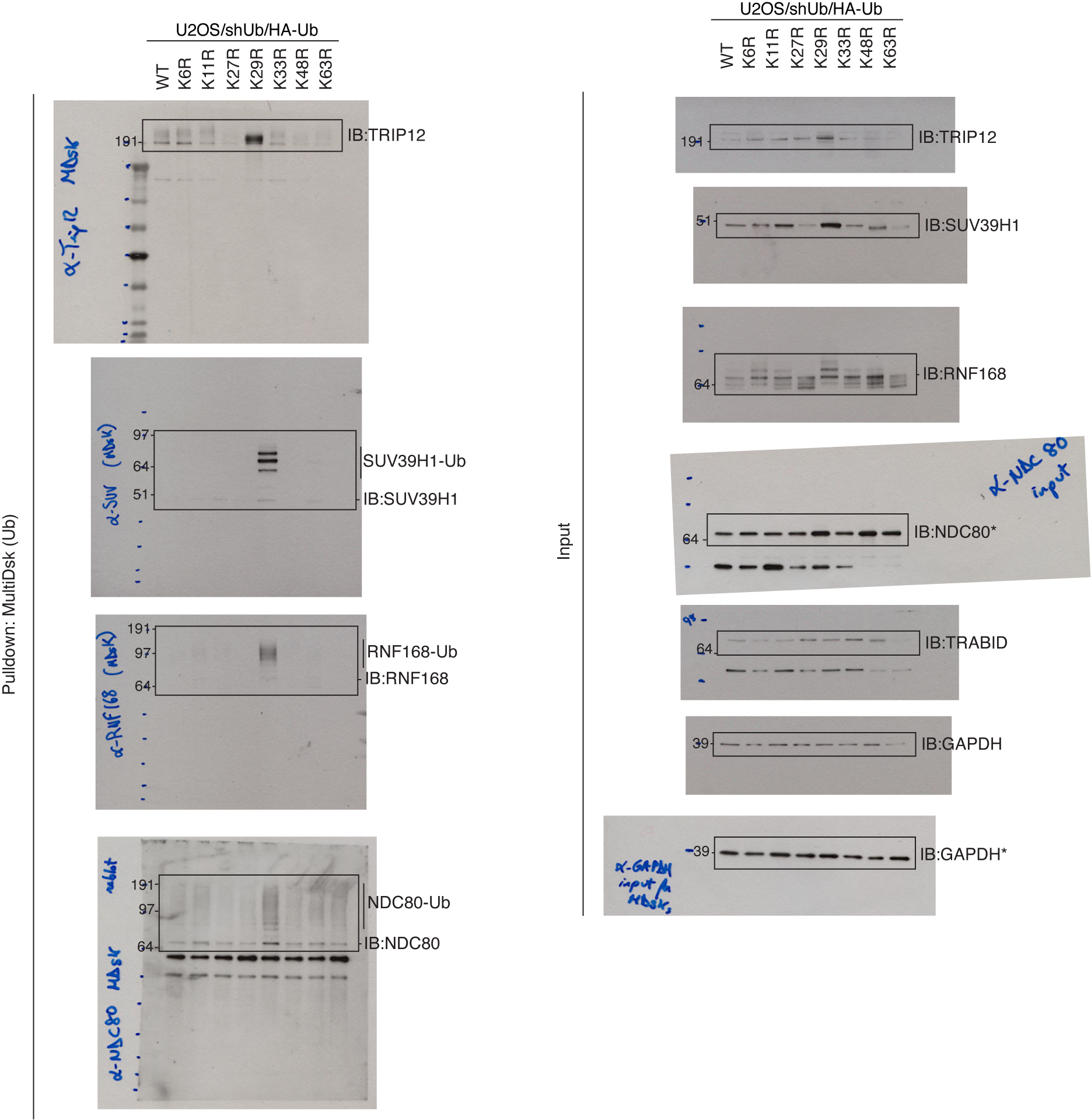

Supplement: Supplementary file 10 — Source data Fig. 3 [file 44318_2025_599_MOESM10_ESM.zip › Figure 3/3A/blots.tif]

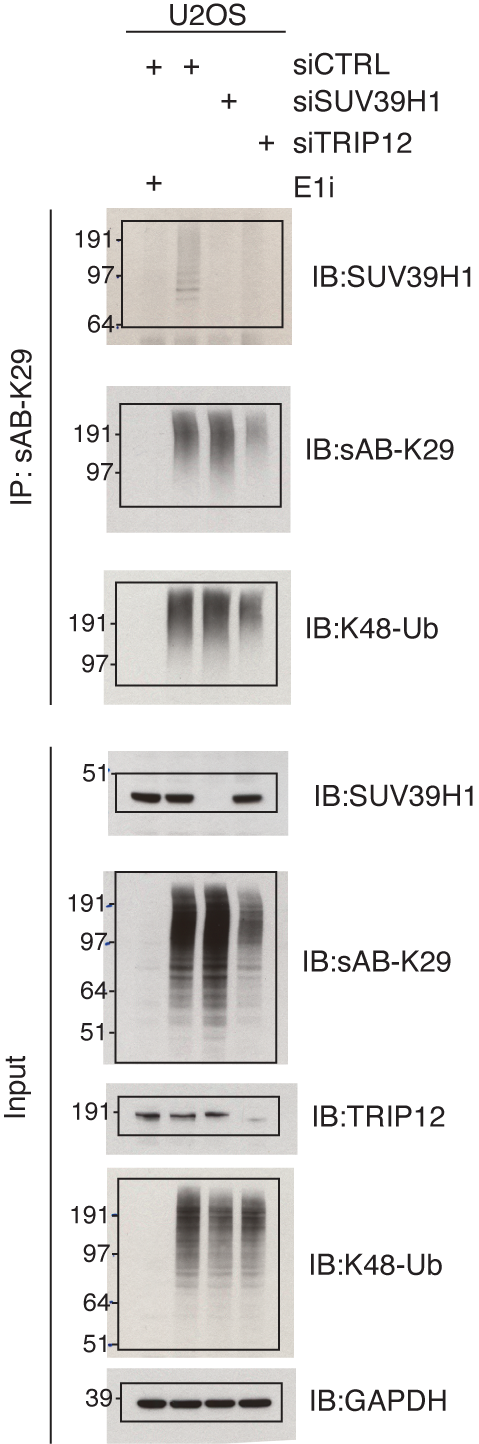

Supplement: Supplementary file 10 — Source data Fig. 3 [file 44318_2025_599_MOESM10_ESM.zip › Figure 3/3F/blots.tif]

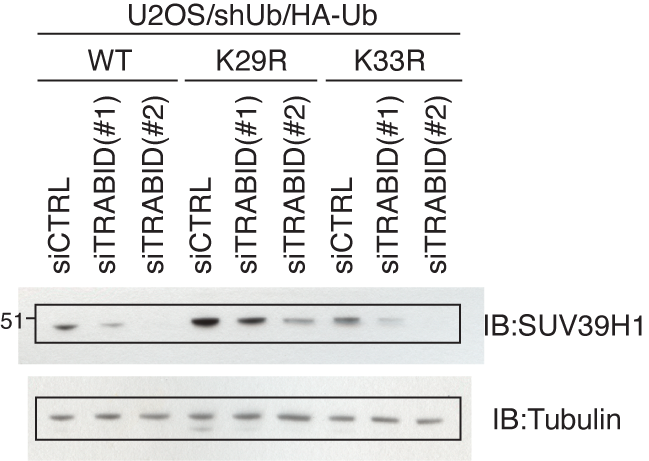

Supplement: Supplementary file 11 — Source data Fig. 4 [file 44318_2025_599_MOESM11_ESM.zip › Figure 4/4E/blots.tif]

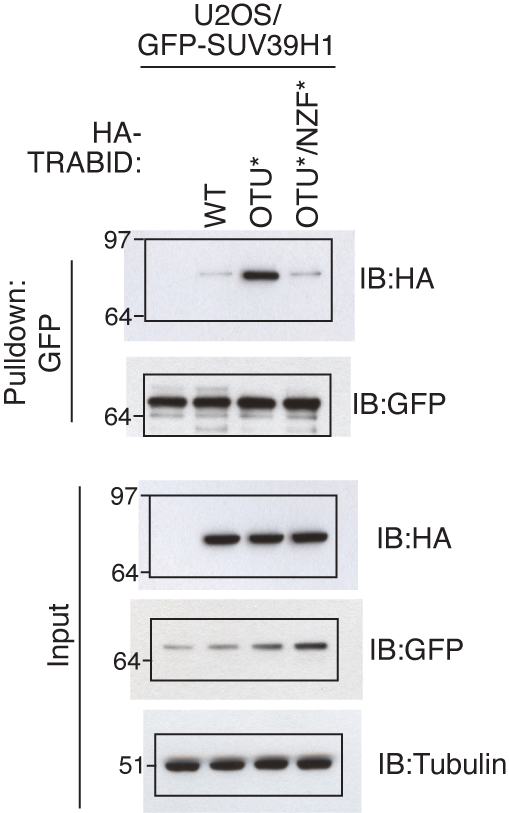

Supplement: Supplementary file 11 — Source data Fig. 4 [file 44318_2025_599_MOESM11_ESM.zip › Figure 4/4B/blots.tif]

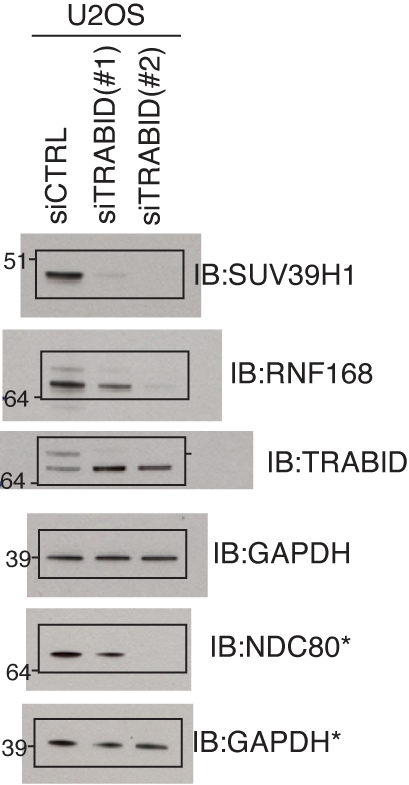

Supplement: Supplementary file 11 — Source data Fig. 4 [file 44318_2025_599_MOESM11_ESM.zip › Figure 4/4C/blots.tif]

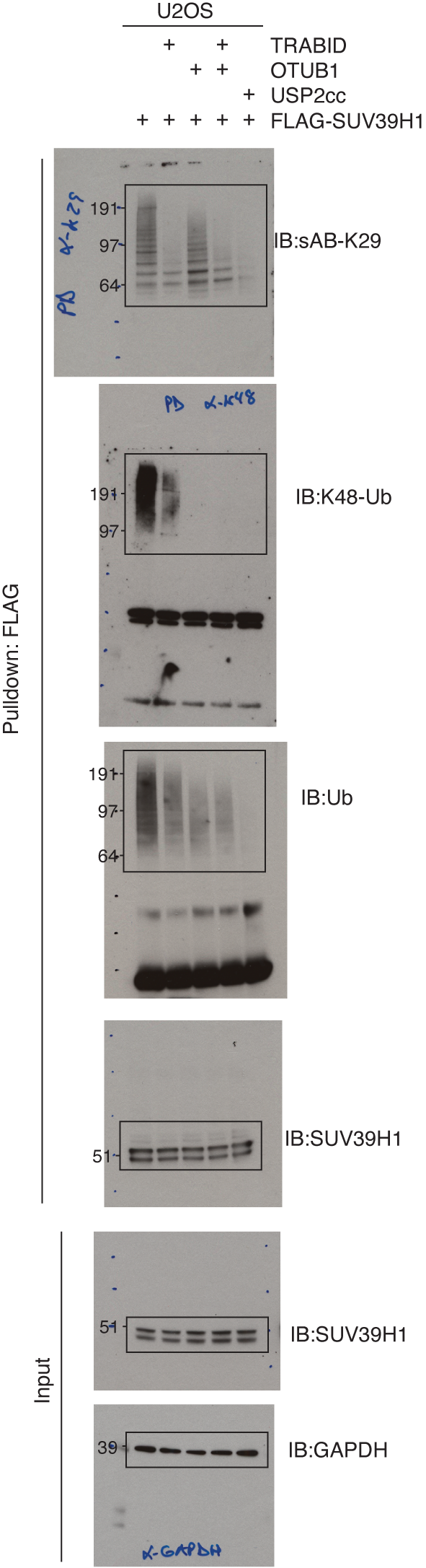

Supplement: Supplementary file 11 — Source data Fig. 4 [file 44318_2025_599_MOESM11_ESM.zip › Figure 4/4F/blots.tif]

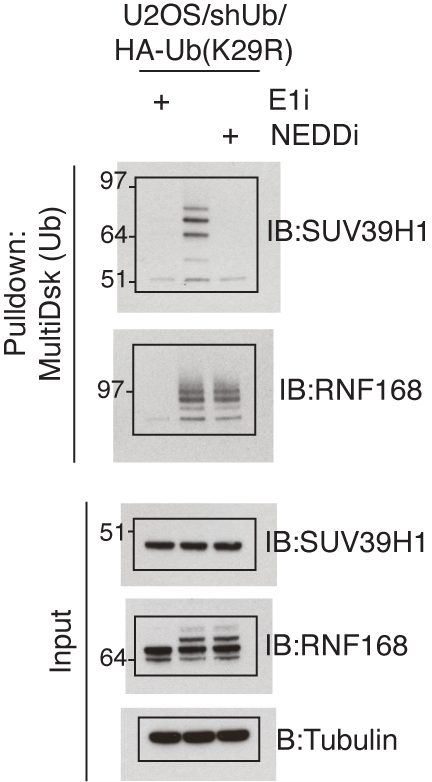

Supplement: Supplementary file 11 — Source data Fig. 4 [file 44318_2025_599_MOESM11_ESM.zip › Figure 4/4H/blots.tif]

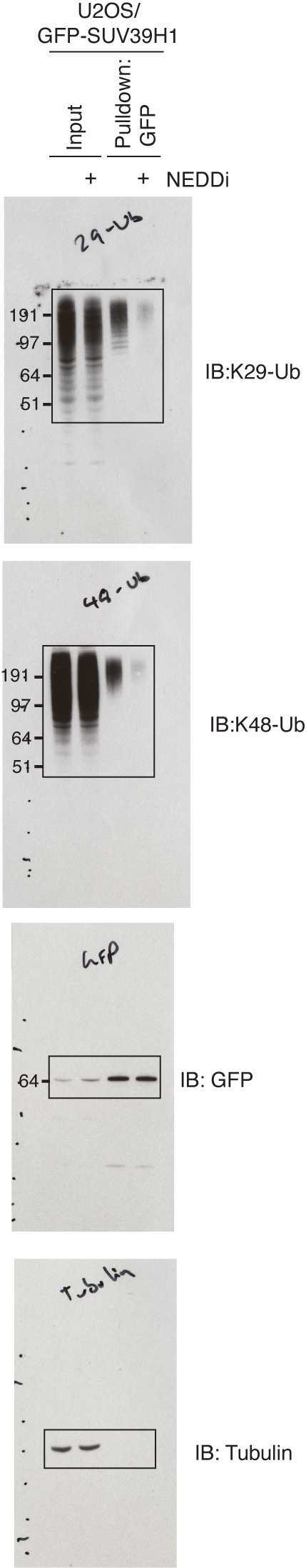

Supplement: Supplementary file 11 — Source data Fig. 4 [file 44318_2025_599_MOESM11_ESM.zip › Figure 4/4I/blots.tif]

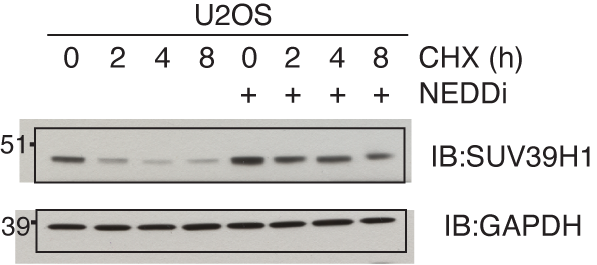

Supplement: Supplementary file 11 — Source data Fig. 4 [file 44318_2025_599_MOESM11_ESM.zip › Figure 4/4G/blots.tif]

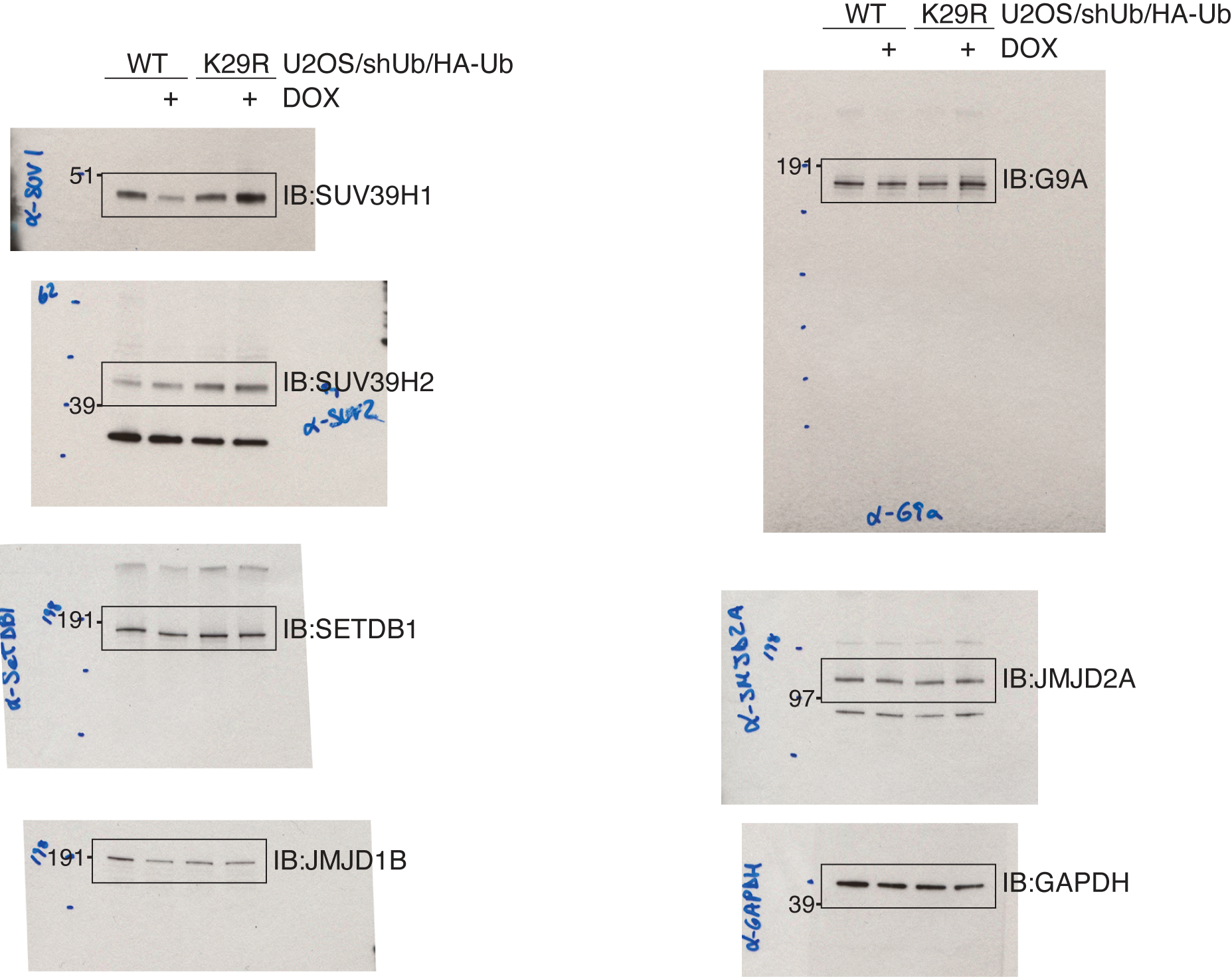

Supplement: Supplementary file 12 — Source data Fig. 5 [file 44318_2025_599_MOESM12_ESM.zip › Figure 5/5B/blots.tif]
