# Supplementary material for: Functional landscape of ubiquitin linkages couples K29-linked ubiquitylation to epigenome integrity
Source: EMBO J. 2025 Oct 22;44(23):6944–78. doi: 10.1038/s44318-025-00599-7 (PMC12669653; doi:10.1038/s44318-025-00599-7)
Supplement: Supplementary file 14 — Expanded View Figures [file 44318_2025_599_MOESM14_ESM.pdf]

Expanded View Figures

Figure EV1. Extended data related to Fig. 1.

(**A**) Immunoblot (IB) analysis of U2OS/shUb and derivative U2OS/shUb/HA-Ub cell lines treated or not with DOX for the indicated times. (**B**) Immunoblot analysis of parental U2OS and derivative U2OS/shUb cell lines. (**C**) U2OS/shUb and derivative replacement cell lines were treated or not with DOX and mRNA levels were analyzed by RT-qPCR. Primers to GAPDH were used as a normalization control (data are technical duplicates of two independent experiments). (**D**) Immunoblot analysis of Ub replacement cell lines treated or not with the BRD4 PROTAC MZ1 (500 nM) overnight. (**E**) Immunoblot analysis of K6-linked Ub chains isolated by pulldown with the K6-Ub binder Biotin-LotA-N in the indicated DOX-treated Ub replacement cell lines exposed to UV-A (500 mJ/cm^2) and/or 4-thiouridine (S4U, 25 mM), combined treatment of which leads to RNA-protein crosslink formation. (**F**) Abundance of K6 diGly peptide after OtUBD (total Ub) enrichment of indicated DOX-treated Ub replacement cell lines in (**E**) (mean ± s.d.; $n = 2$ technical replicates). (**G**) Cell cycle analysis of indicated Ub replacement cell lines by QIBC. All Ub replacement cell lines were treated with DOX for 72 h, except Ub(K48R) cells that were treated for 48 h. Upper panel shows cell cycle fraction (mean ± s.e.m.; $n = 4$ independent experiments; >1000 cells analyzed per condition). Lower panel shows mitotic fraction as determined by QIBC of H3-pS10 immunostaining (mean ± s.e.m.; $n = 7$ independent experiments; >1000 cells analyzed per condition). Source data are available online for this figure.

© The Author(s)

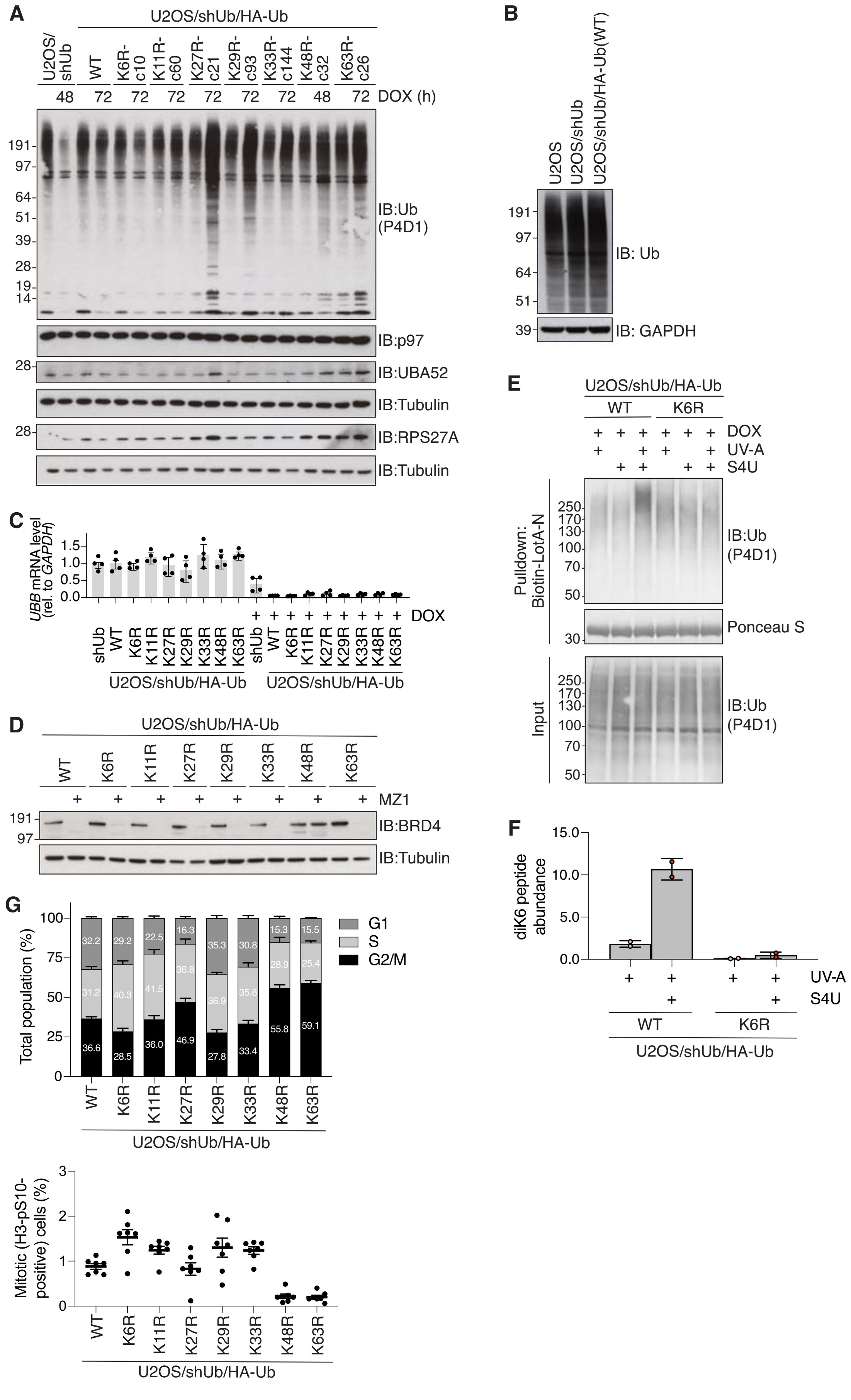

A
U2OS/shUb/HA-Ub
U2OS/shUb
WT
K6R-c10
K11R-c60
K27R-c21
K29R-c93
K33R-c144
K48R-c32
K63R-c26
48 72 72 72 72 72 72 48 72 DOX (h)
191
97
64
51
39
28
19
14
IB:Ub (P4D1)
IB:p97
IB:UBA52
IB:Tubulin
IB:RPS27A
IB:Tubulin
B
U2OS
U2OS/shUb
U2OS/shUb/HA-Ub(WT)
IB: Ub
IB: GAPDH
C
UBB mRNA level (rel. to GAPDH)
DOX
shUb WT K6R K11R K27R K29R K33R K48R K63R
U2OS/shUb/HA-Ub
D
U2OS/shUb/HA-Ub
WT K6R K11R K27R K29R K33R K48R K63R
MZ1
IB:BRD4
IB:Tubulin
E
U2OS/shUb/HA-Ub
WT K6R
DOX
UV-A
S4U
Pulldown: Biotin-LotA-N
IB:Ub (P4D1)
Ponceau S
Input
IB:Ub (P4D1)
F
diK6 peptide abundance
UV-A
S4U
WT K6R
U2OS/shUb/HA-Ub
G
Total population (%)
G1
S
G2/M
WT K6R K11R K27R K29R K33R K48R K63R
U2OS/shUb/HA-Ub
Mitotic (H3-pS10-positive) cells (%)
WT K6R K11R K27R K29R K33R K48R K63R
U2OS/shUb/HA-Ub

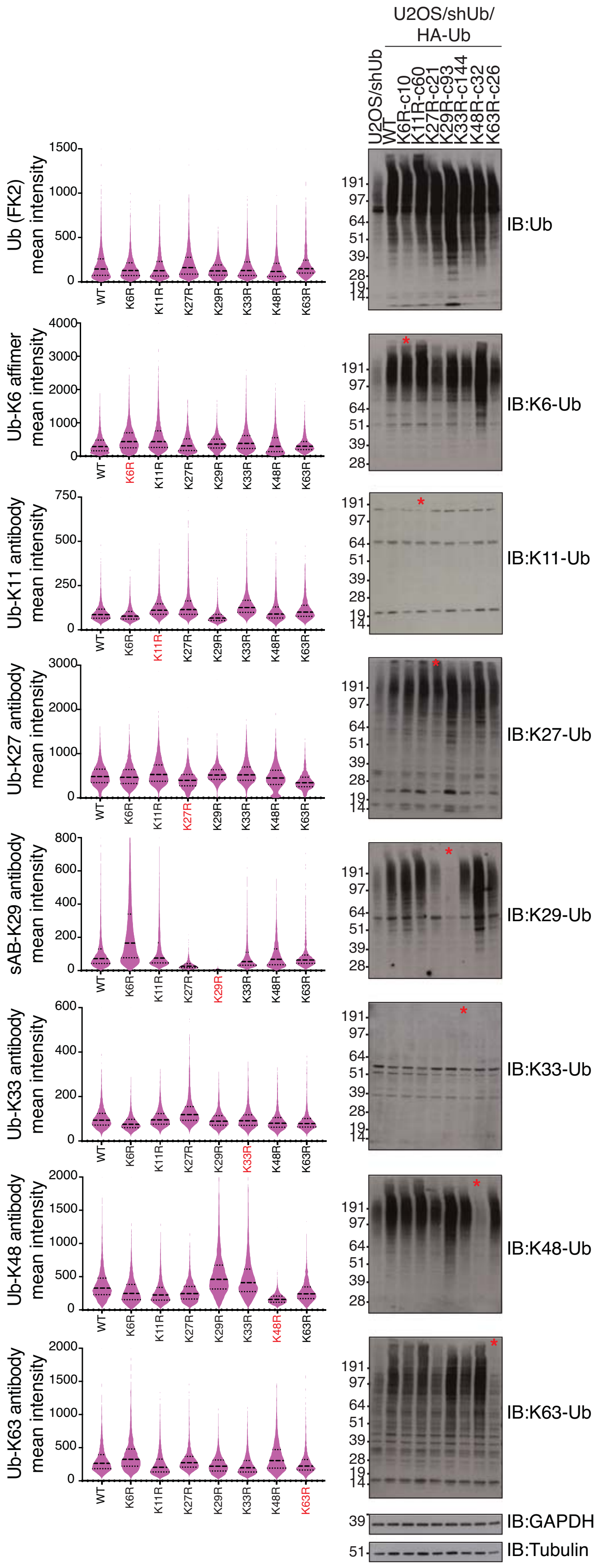
U2OS/shUb/
HA-Ub
U2OS/shUb
WT
K6R-c10
K11R-c60
K27R-c21
K29R-c93
K33R-c144
K48R-c32
K63R-c26
Ub (FK2) mean intensity
IB:Ub
Ub-K6 affimer mean intensity
IB:K6-Ub
Ub-K11 antibody mean intensity
IB:K11-Ub
Ub-K27 antibody mean intensity
IB:K27-Ub
sAB-K29 antibody mean intensity
IB:K29-Ub
Ub-K33 antibody mean intensity
IB:K33-Ub
Ub-K48 antibody mean intensity
IB:K48-Ub
Ub-K63 antibody mean intensity
IB:K63-Ub
IB:GAPDH
IB:Tubulin
WT
K6R
K11R
K27R
K29R
K33R
K48R
K63R

 © The Author(s)

Figure EV2. Extended data related to Fig. 2.

QIBC analysis (left) and immunoblot analysis (right) of DOX-treated Ub replacement cell lines using the indicated linkage-specific antibodies or binders (thick dashed lines, median; dotted lines, quartiles). Data were a single representative replicate from three independent experiments. Source data are available online for this figure.

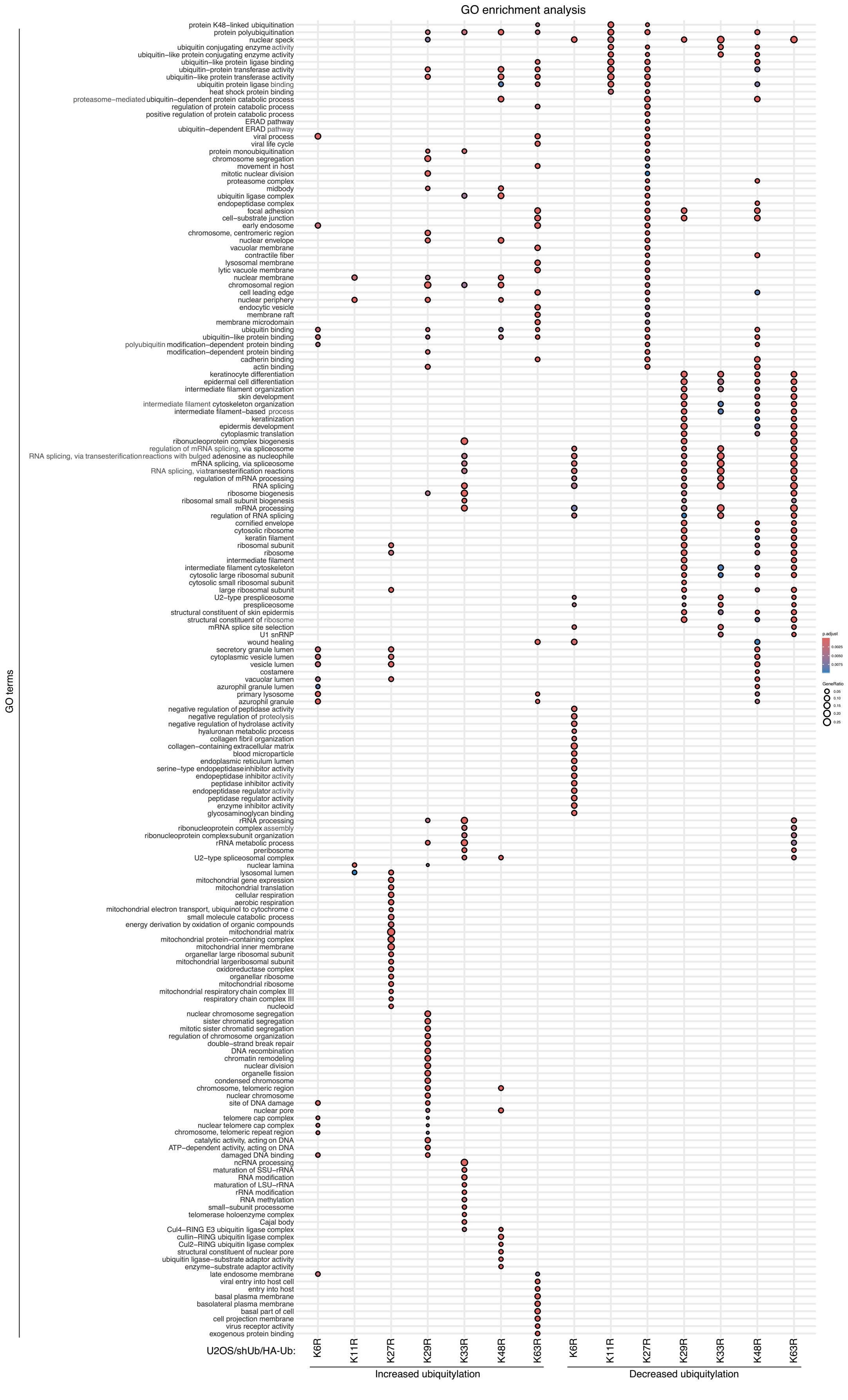
GO enrichment analysis
GO terms
protein K48−linked ubiquitination
protein polyubiquitination
nuclear speck
ubiquitin conjugating enzyme activity
ubiquitin−like protein conjugating enzyme activity
ubiquitin−like protein ligase binding
ubiquitin−protein transferase activity
ubiquitin−like protein transferase activity
ubiquitin protein ligase binding
heat shock protein binding
proteasome−mediated ubiquitin−dependent protein catabolic process
regulation of protein catabolic process
positive regulation of protein catabolic process
ERAD pathway
ubiquitin−dependent ERAD pathway
viral process
viral life cycle
protein monoubiquitination
chromosome segregation
movement in host
mitotic nuclear division
proteasome complex
midbody
ubiquitin ligase complex
endopeptidase complex
focal adhesion
cell−substrate junction
early endosome
chromosome, centromeric region
nuclear envelope
vacuolar membrane
contractile fiber
lysosomal membrane
lytic vacuole membrane
nuclear membrane
chromosomal region
cell leading edge
nuclear periphery
endocytic vesicle
membrane raft
membrane microdomain
ubiquitin binding
ubiquitin−like protein binding
polyubiquitin modification−dependent protein binding
modification−dependent protein binding
cadherin binding
actin binding
keratinocyte differentiation
epidermal cell differentiation
intermediate filament organization
skin development
intermediate filament cytoskeleton organization
intermediate filament−based process
keratinization
epidermis development
cytoplasmic translation
ribonucleoprotein complex biogenesis
regulation of mRNA splicing, via spliceosome
RNA splicing, via transesterification reactions with bulged adenosine as nucleophile
mRNA splicing, via spliceosome
RNA splicing, via transesterification reactions
regulation of mRNA processing
RNA splicing
ribosome biogenesis
ribosomal small subunit biogenesis
mRNA processing
regulation of RNA splicing
cornified envelope
cytosolic ribosome
keratin filament
ribosomal subunit
ribosome
intermediate filament
intermediate filament cytoskeleton
cytosolic large ribosomal subunit
cytosolic small ribosomal subunit
large ribosomal subunit
U2−type prespliceosome
prespliceosome
structural constituent of skin epidermis
structural constituent of ribosome
mRNA splice site selection
U1 snRNP
wound healing
secretory granule lumen
cytoplasmic vesicle lumen
vesicle lumen
costamere
vacuolar lumen
azurophil granule lumen
primary lysosome
azurophil granule
negative regulation of peptidase activity
negative regulation of proteolysis
negative regulation of hydrolase activity
hyaluronan metabolic process
collagen fibril organization
collagen−containing extracellular matrix
blood microparticle
endoplasmic reticulum lumen
serine−type endopeptidase inhibitor activity
endopeptidase inhibitor activity
peptidase inhibitor activity
endopeptidase regulator activity
peptidase regulator activity
enzyme inhibitor activity
glycosaminoglycan binding
rRNA processing
ribonucleoprotein complex assembly
ribonucleoprotein complex subunit organization
rRNA metabolic process
preribosome
U2−type spliceosomal complex
nuclear lamina
lysosomal lumen
mitochondrial gene expression
mitochondrial translation
cellular respiration
aerobic respiration
mitochondrial electron transport, ubiquinol to cytochrome c
small molecule catabolic process
energy derivation by oxidation of organic compounds
mitochondrial matrix
mitochondrial protein−containing complex
mitochondrial inner membrane
organellar large ribosomal subunit
mitochondrial large ribosomal subunit
oxidoreductase complex
organellar ribosome
mitochondrial ribosome
mitochondrial respiratory chain complex III
respiratory chain complex III
nucleoid
nuclear chromosome segregation
sister chromatid segregation
mitotic sister chromatid segregation
regulation of chromosome organization
double−strand break repair
DNA recombination
chromatin remodeling
nuclear division
organelle fission
condensed chromosome
chromosome, telomeric region
nuclear chromosome
site of DNA damage
nuclear pore
telomere cap complex
nuclear telomere cap complex
chromosome, telomeric repeat region
catalytic activity, acting on DNA
ATP−dependent activity, acting on DNA
damaged DNA binding
ncRNA processing
maturation of SSU−rRNA
RNA modification
maturation of LSU−rRNA
rRNA modification
RNA methylation
small−subunit processome
telomerase holoenzyme complex
Cajal body
Cul4−RING E3 ubiquitin ligase complex
cullin−RING ubiquitin ligase complex
Cul2−RING ubiquitin ligase complex
structural constituent of nuclear pore
ubiquitin ligase−substrate adaptor activity
enzyme−substrate adaptor activity
late endosome membrane
viral entry into host cell
entry into host
basal plasma membrane
basolateral plasma membrane
basal part of cell
cell projection membrane
virus receptor activity
exogenous protein binding
U2OS/shUb/HA-Ub:
K6R
K11R
K27R
K29R
K33R
K48R
K63R
K6R
K11R
K27R
K29R
K33R
K48R
K63R
Increased ubiquitylation
Decreased ubiquitylation
p.adjust
0.0025
0.0050
0.0075
GeneRatio
0.05
0.10
0.15
0.20
0.25

Figure EV3. Extended data related to Fig. 2.

GO term analysis of cellular compartments enriched among proteins showing significantly up- or downregulated ubiquitylation in Ub(K-to-R)-replaced cells relative to Ub(WT)-replaced cells (Fig. 2B–H) ($n = 3$ technical replicates; unpaired two-tailed *t*-test, Benjamini–Hochberg corrected).

© The Author(s)

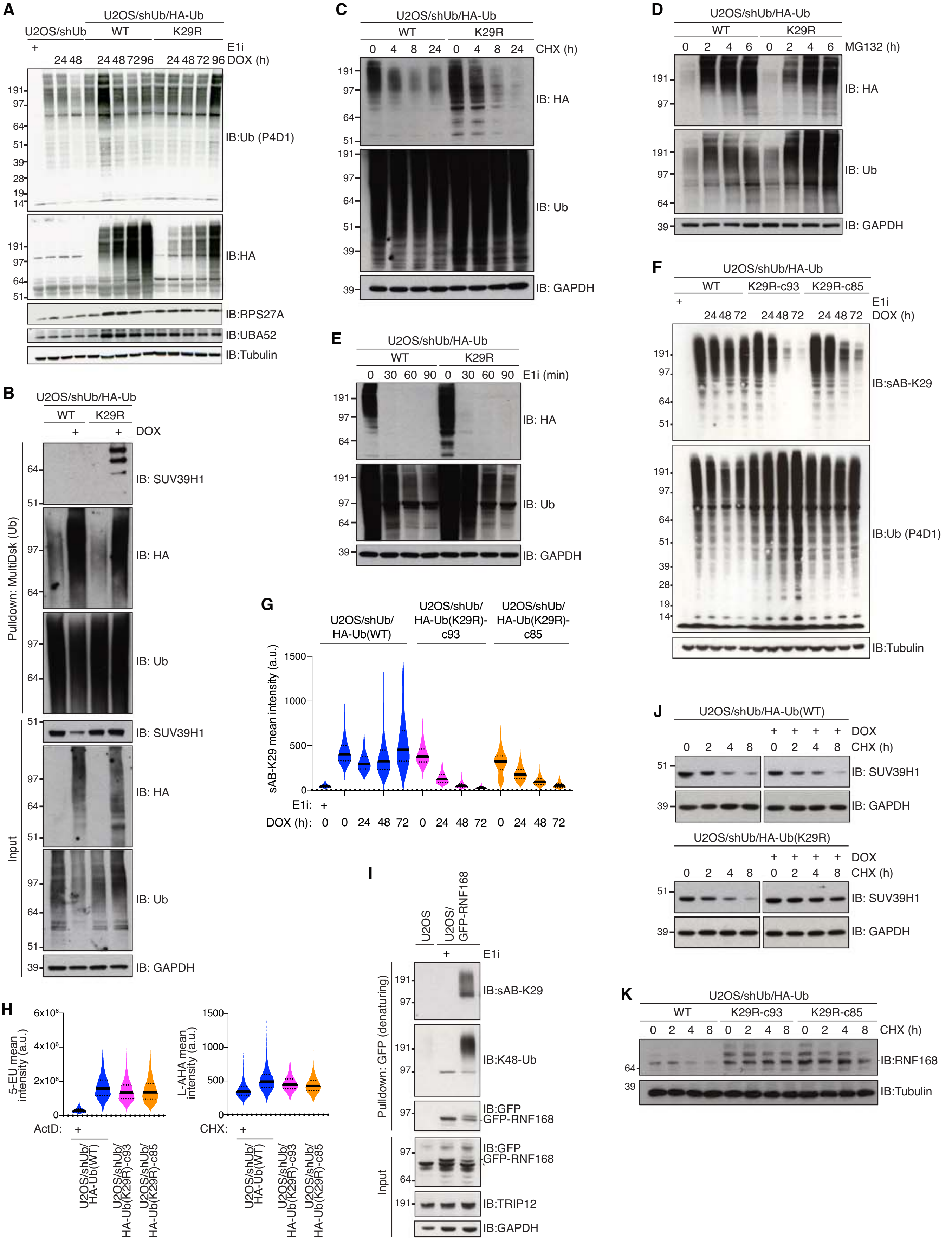
A
U2OS/shUb/HA-Ub
U2OS/shUb
WT
K29R
E1i
DOX (h)
24 48
24 48 72 96
24 48 72 96
IB:Ub (P4D1)
IB:HA
IB:RPS27A
IB:UBA52
IB:Tubulin
B
U2OS/shUb/HA-Ub
WT
K29R
DOX
Pulldown: MultiDsk (Ub)
Input
IB: SUV39H1
IB: HA
IB: Ub
IB: GAPDH
C
U2OS/shUb/HA-Ub
WT
K29R
0 4 8 24 0 4 8 24
CHX (h)
IB: HA
IB: Ub
IB: GAPDH
D
U2OS/shUb/HA-Ub
WT
K29R
0 2 4 6 0 2 4 6
MG132 (h)
IB: HA
IB: Ub
IB: GAPDH
E
U2OS/shUb/HA-Ub
WT
K29R
0 30 60 90 0 30 60 90
E1i (min)
IB: HA
IB: Ub
IB: GAPDH
F
U2OS/shUb/HA-Ub
WT
K29R-c93
K29R-c85
E1i
DOX (h)
24 48 72
24 48 72
24 48 72
IB:sAB-K29
IB:Ub (P4D1)
IB:Tubulin
G
U2OS/shUb/HA-Ub(WT)
U2OS/shUb/HA-Ub(K29R)-c93
U2OS/shUb/HA-Ub(K29R)-c85
sAB-K29 mean intensity (a.u.)
E1i:
DOX (h):
0 0 24 48 72 0 24 48 72 0 24 48 72
H
5-EU mean intensity (a.u.)
ActD:
L-AHA mean intensity (a.u.)
CHX:
U2OS/shUb/HA-Ub(WT)
U2OS/shUb/HA-Ub(K29R)-c93
U2OS/shUb/HA-Ub(K29R)-c85
I
U2OS
U2OS/GFP-RNF168
E1i
Pulldown: GFP (denaturing)
Input
IB:sAB-K29
IB:K48-Ub
IB:GFP
GFP-RNF168
IB:TRIP12
IB:GAPDH
J
U2OS/shUb/HA-Ub(WT)
U2OS/shUb/HA-Ub(K29R)
DOX
CHX (h)
0 2 4 8 0 2 4 8
IB: SUV39H1
IB: GAPDH
K
U2OS/shUb/HA-Ub
WT
K29R-c93
K29R-c85
0 2 4 8 0 2 4 8 0 2 4 8
CHX (h)
IB:RNF168
IB:Tubulin

 © The Author(s)

Figure EV4. Extended data related to Fig. 3. (**A**) Immunoblot analysis of U2OS/shUb, U2OS/shUb/HA-Ub(WT) and U2OS/shUb/HA-Ub(K29R)-c93 cell lines treated or not with DOX for the indicated times. Where indicated, cells were treated with Ub E1 inhibitor (E1i) for 1 h. (**B**) Immunoblot analysis of Ub conjugates isolated via MultiDsk pulldown from indicated Ub replacement cell lines treated or not with DOX. (**C**) Immunoblot analysis of DOX-treated Ub(WT) and Ub(K29R) cell lines treated or not with cycloheximide (CHX) for the indicated times. (**D**) As in (**C**), except that cells were treated with MG132 for the indicated times. (**E**) As in (**C**), except that cells were treated with MLN-7243 (E1i) for the indicated times. (**F**) As in (**A**), but using Ub(WT), Ub(K29R)-c93 and Ub(K29R)-c85 replacement cell lines. (**G**) QIBC analysis of Ub(WT) and Ub(K29R) replacement cell lines treated with DOX for the indicated times and immunostained with sAB-K29 (thick dashed line, median; dotted lines, quartiles). Data were a single representative replicate from three independent experiments (>1000 cells analyzed per sample). (**H**) QIBC analysis of DOX-treated Ub(WT) and Ub(K29R) replacement cell lines treated or not with the transcription inhibitor Actinomycin D (ActD) or the protein synthesis inhibitor cycloheximide (CHX), where indicated and stained for nascent RNA (upper) or nascent protein (lower panel) by detection of incorporated EU and AHA, respectively (thick lines, median; dotted lines, quartiles). Data were a single representative replicate from three independent experiments. (**I**) U2OS cells or a derivative cell line stably expressing GFP-RNF168 were treated or not with Ub E1i, subjected to GFP pulldown under denaturing conditions and immunoblotted with the indicated antibodies. (**J**) Immunoblot analysis of Ub(WT) and Ub(K29R) cell lines incubated or not with DOX for 72 h and treated with cycloheximide (CHX) for the indicated times. (**K**) Immunoblot analysis of DOX-treated Ub(WT) and Ub(K29R) cell lines treated or not with cycloheximide (CHX) for the indicated times. Source data are available online for this figure.

© The Author(s)

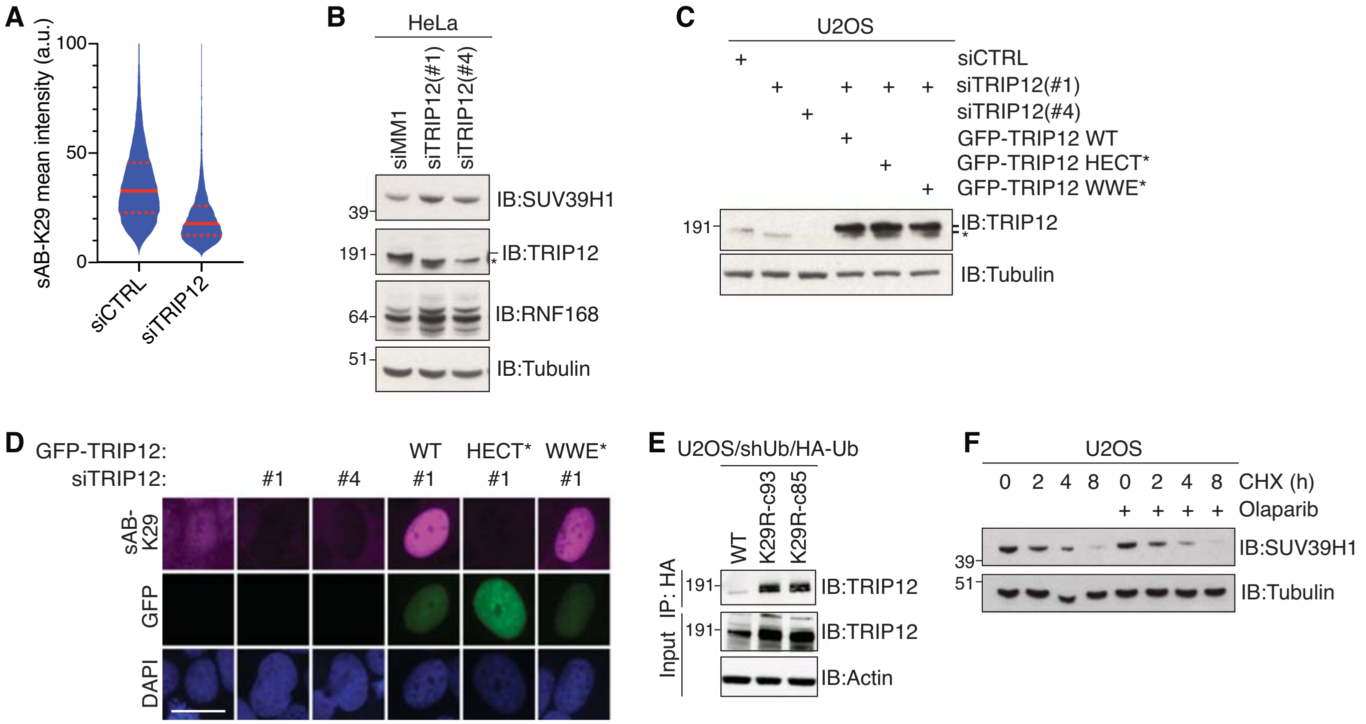

Figure EV5. Extended data related to Fig. 3.

(**A**) QIBC analysis of HeLa cells transfected with indicated siRNAs (thick line, median; dotted lines, quartiles). Data were a single representative replicate from three independent experiments (>1000 cells analyzed per sample). (**B**) Immunoblot of HeLa cells transfected with indicated siRNAs. (**C**) Immunoblot analysis of U2OS cells sequentially transfected with indicated siRNA and siRNA-resistant GFP-TRIP12 expression plasmids. (**D**) Representative images of individual cells from data shown in Fig. 3J. Scale bar, 25 µm. (**E**) DOX-treated Ub(WT) and Ub(K29R) replacement cell lines were subjected to HA IP under denaturing conditions and immunoblotted with the indicated antibodies. (**F**) Immunoblot analysis of SUV39H1 in U2OS cells treated or not with cycloheximide (CHX) for the indicated times in the absence or presence of the PARP1 inhibitor Olaparib. Source data are available online for this figure.

 © The Author(s)

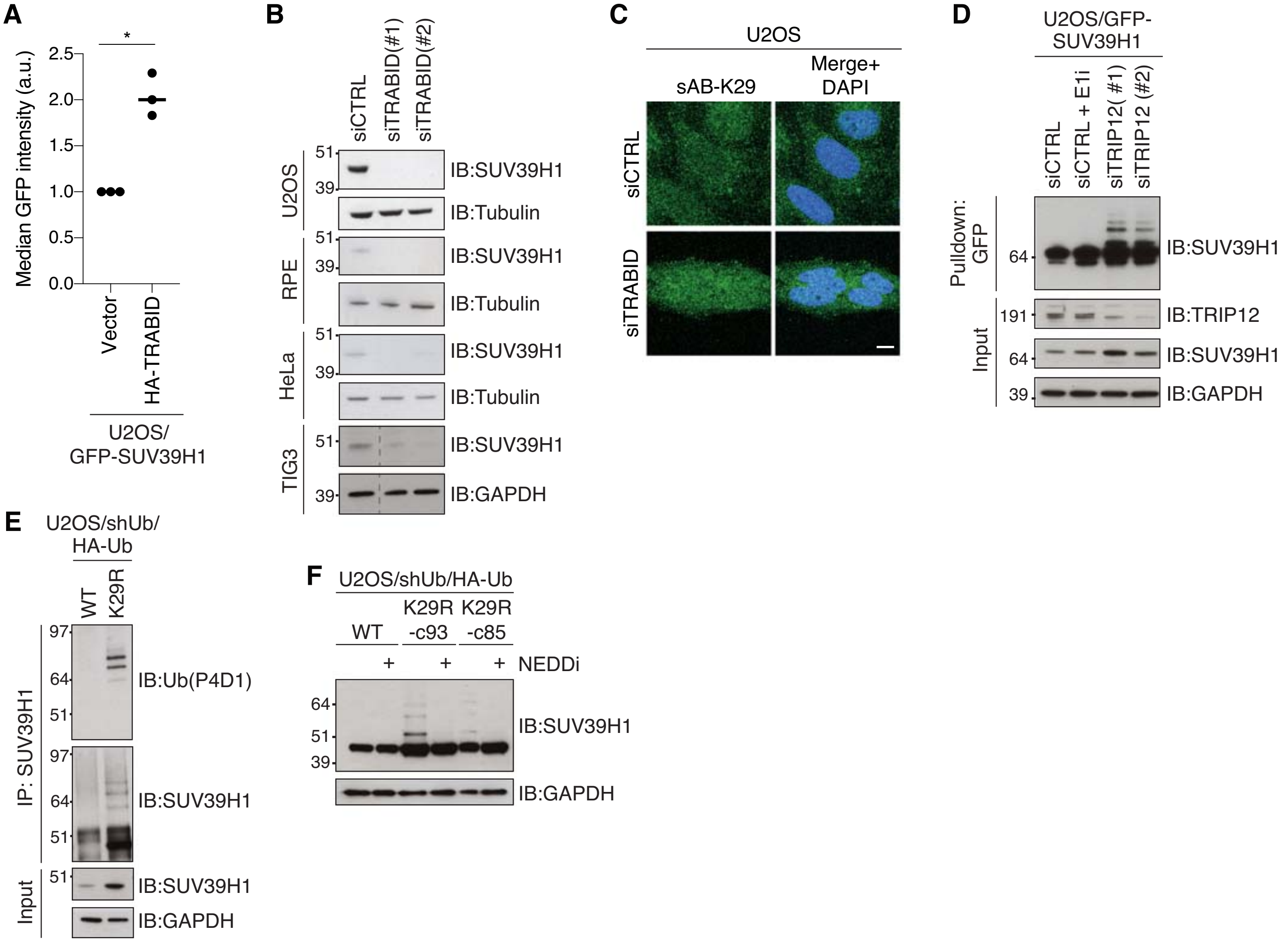

Figure EV6. Extended data related to Fig. 4.

(**A**) QIBC analysis of U2OS/GFP-SUV39H1 cells transfected or not with HA-TRABID expression construct. Data shown is fold change of mean of average nuclear GFP intensity relative to untransfected cells ($n = 3$ independent experiments; $*p < 0.05$, two-tailed t-test). (**B**) Immunoblot analysis of indicated cell lines transfected with non-targeting control (CTRL) or TRABID siRNAs. (**C**) Representative images of U2OS cells transfected with the indicated siRNAs and immunostained with sAB-K29. (**D**) U2OS/GFP-SUV39H1 cells transfected with siRNAs and treated with Ub E1 inhibitor, where indicated, were subjected to GFP pulldown under denaturing conditions and immunoblotted with indicated antibodies. (**E**) DOX-treated Ub(WT) and Ub(K29R) replacement cell lines were subjected to SUV39H1 IP and immunoblotted with indicated antibodies. (**F**) Immunoblot analysis of DOX-treated Ub(WT) and Ub(K29R) replacement cell lines treated or not with NEDDylation inhibitor for 4 h. Source data are available online for this figure.

© The Author(s)

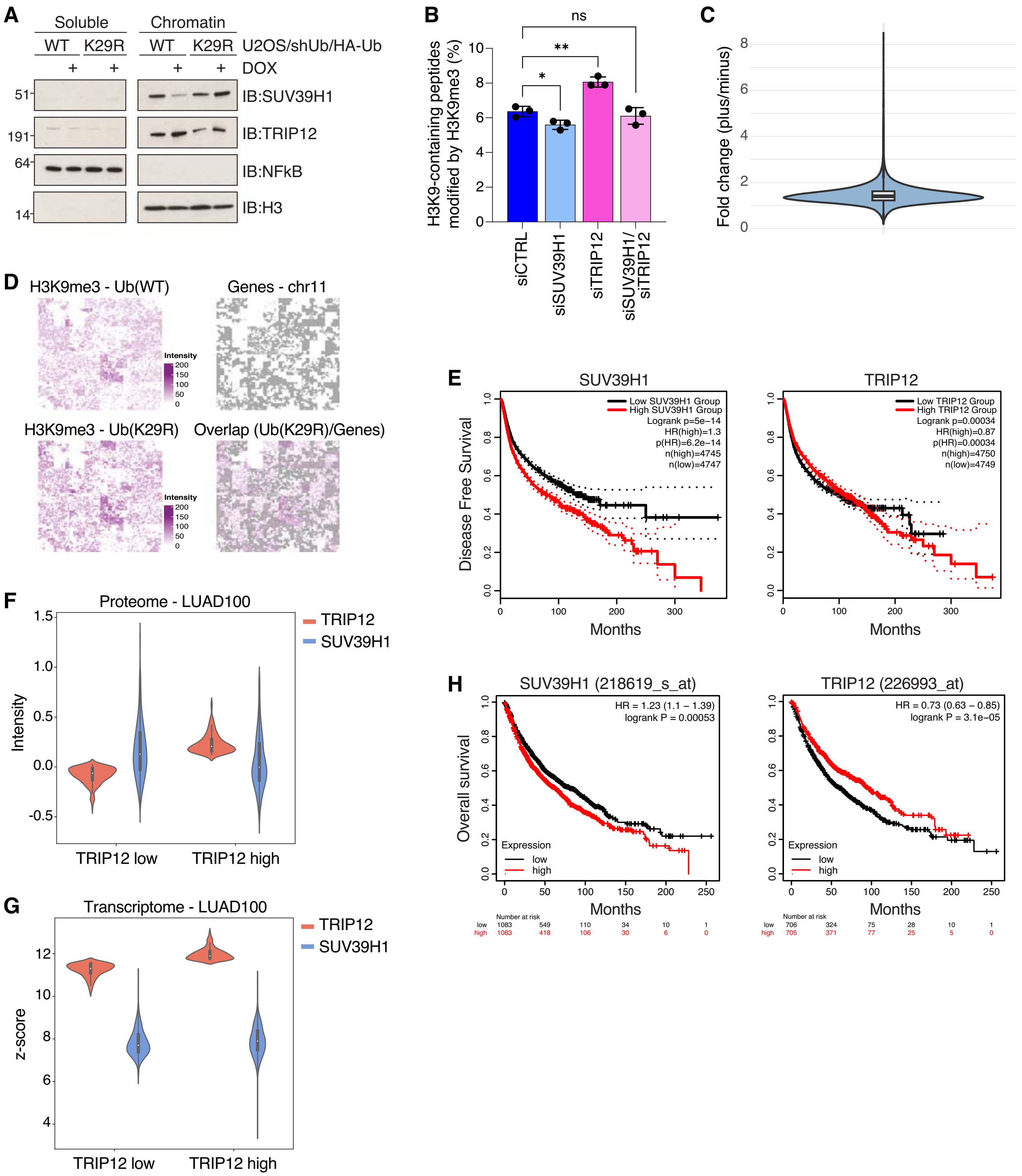
A
Soluble
Chromatin
WT K29R WT K29R U2OS/shUb/HA-Ub
+ + + + DOX
51
191
64
14
IB:SUV39H1
IB:TRIP12
IB:NFkB
IB:H3
B
ns
**
*
H3K9-containing peptides modified by H3K9me3 (%)
siCTRL
siSUV39H1
siTRIP12
siSUV39H1/ siTRIP12
C
Fold change (plus/minus)
D
H3K9me3 - Ub(WT)
Genes - chr11
H3K9me3 - Ub(K29R)
Overlap (Ub(K29R)/Genes)
Intensity
200
150
100
50
0
E
SUV39H1
TRIP12
Disease Free Survival
Low SUV39H1 Group
High SUV39H1 Group
Logrank p=5e−14
HR(high)=1.3
p(HR)=6.2e−14
n(high)=4745
n(low)=4747
Low TRIP12 Group
High TRIP12 Group
Logrank p=0.00034
HR(high)=0.87
p(HR)=0.00034
n(high)=4750
n(low)=4749
Months
F
Proteome - LUAD100
Intensity
TRIP12
SUV39H1
TRIP12 low
TRIP12 high
G
Transcriptome - LUAD100
z-score
TRIP12
SUV39H1
TRIP12 low
TRIP12 high
H
SUV39H1 (218619_s_at)
TRIP12 (226993_at)
Overall survival
HR = 1.23 (1.1 − 1.39)
logrank P = 0.00053
HR = 0.73 (0.63 − 0.85)
logrank P = 3.1e−05
Expression
low
high
Months
Number at risk
low 1083 549 110 34 10 1
high 1083 418 106 30 6 0
low 706 324 75 28 10 1
high 705 371 77 25 5 0

 © The Author(s)

Figure EV7. Extended data related to Fig. 5.

(**A**) Immunoblot analysis of soluble and chromatin-enriched fractions of Ub(WT) and Ub(K29R) replacement cell lines treated or not with DOX. (**B**) Quantitative proteomic analysis of H3K9me3 abundance (H3K9me3 peptides unmodified at K14) in U2OS cells treated with indicated siRNAs ($n = 3$ biological replicates; $^{**}p < 0.01$; $^{*}p < 0.05$; ns non-significant, unpaired two-tailed *t*-test). (**C**) Violin plot showing genome-wide distribution of H3K9me3 fold change values per 10 kb genomic bin, calculated from spike-in normalized H3K9me3 ChIP-seq signal in Ub(K29R)-replaced cells (+DOX) relative to non-replaced Ub(K29R) cells (−DOX). Each point represents a non-overlapping genomic bin, and fold changes were computed as the ratio of normalized signal between the two conditions. (**D**) Hilbert analysis depicting the spatial localization of H3K9me3 across chromosome 11 in DOX-treated Ub(WT) and Ub(K29R) replacement cell lines. Overlap panel depicts the signal from Ub(K29R) cells over genes on chromosome 11. (**E**) Kaplan–Meier analysis of disease-free survival stratified by expression of SUV39H1 (left) or TRIP12 (right) in a pan-cancer cohort. Dotted lines represent a 95% confidence interval. Analysis was performed using GEPIA2 on 33 cancer types. Hypothesis evaluation was performed with the log-rank test. Hazard ratios were calculated according to the Cox proportional-hazards model. (**F**) LUAD-100 proteome levels of indicated proteins stratified by relatively high and low TRIP12-expressing samples. Data were shown as the log2 ratio of reporter ion intensity. (**G**) LUAD-100 transcriptome levels stratified by relatively high and low TRIP12-expressing samples. (**H**) Kaplan–Meier analysis of overall survival in non-small cell lung cancer (NSCLC) stratified on SUV39H1 (left) or TRIP12 (right) expression. Analysis was performed using the KMPlotter tool. Hazard ratios and *p* values were calculated through Cox regression. Source data are available online for this figure.

© The Author(s)
